# Expression Profiling of Human Basophils: Modulation by Cytokines and Secretagogues

**DOI:** 10.1371/journal.pone.0126435

**Published:** 2015-05-11

**Authors:** Donald MacGlashan

**Affiliations:** Johns Hopkins Asthma and Allergy Center, Baltimore, MD, United States of America; Friedrich-Alexander-University Erlangen, GERMANY

## Abstract

Human basophils are an accessible participant of the human allergic reaction. There is natural variation in various functional endpoints and in signaling molecule expression but there has been only a limited effort to place this information in the context of mRNA expression profiles. This study examined the hypothesis that unique mRNA signatures could be identified during the response of human basophils to several known forms of stimulation. Highly purified human basophils were cultured in vitro and exposed to IL-3, IL-5, NGF, IL-33, IL-2, anti-IgE Ab, or FMLP and the mRNA profiles examined by microarrays. The response to IL-3 and anti-IgE Ab were examined on 2–3 time frames and the response to IL-3 examined at several concentrations. In addition, the mRNA signatures of 3 different potential phenotypes were examined. These included basophils with the so-called non-releaser phenotype, and basophils from atopic and non-atopic subjects. Given the role of IL-3 in basophil maturation and the known profound effects on mature basophil function, it was not surprising that IL-3 showed the greatest influence on the basophil transcriptome. However, it also became apparent that the act of isolating and culturing basophils was sufficient to induce a large number of changes in the transcriptome, despite high viability and recovery. These “culture-effect” changes dominated the changes in mRNA profiles induced by other stimuli. Unique signatures for anti-IgE antibody and IL-33 could be identified although the number of gene transcripts (6–30) that were unique to these two stimuli was very limited. There were no apparent unique profiles for IL-5, NGF, IL-2 or FMLP. Therefore, a potential tool for screening basophil phenotypes was limited to changes that could be induced by IL-3 (or no IL-3), IL-33 and anti-IgE Ab.

## Introduction

Allergic diseases result from an immune response that is characterized by the presence and activities of IgE antibody. The basophil is one of the target cells that has been shown in a variety of studies to participate in allergic diseases through its ability to bind IgE. Many studies have shown that there is significant variability in the ability of a subject’s basophils to respond through the IgE pathway. Naturally, there is variability due to the relative presence of IgE and in particular, antigen-specific IgE. But it is also clear that there is considerable variability in the cell’s intrinsic sensitivity to IgE-mediated stimulation. For example, numerous studies have noted that stimulating basophils with anti-IgE generates a histamine release response that varies from 0 to 100% and a given individual’s ability to respond at a particular level is a long-lasting attribute [1,2]. A limited series of signal transduction studies have also shown that expression of some signaling proteins are either under tight control or widely variable [3,4]. Basophils have also been found to display functional phenotypes that track with certain diseases. For example, one of the earliest functional behaviors identified for basophils was an increase in the spontaneous release from these cells once isolated by a variety of standard methodologies [5–8]. It is generally felt that spontaneous release is not occurring *in vivo* but an unknown state of the cell establishes a condition such that during isolation it begins secreting in simple calcium-containing buffers. The basis for this cellular state is unknown but the spontaneous release behavior has been associated with atopy and asthma or with food allergies [5–7,9,10]. In a subpopulation of patients with chronic idiopathic urticaria, one finds basophils whose responsiveness to IgE-mediated stimulation is blunted [11–15]. It has been proposed that changes in the expression of SHIP (SH2-containing inositol 5’ phosphatase) is associated with poor release from this type of basophil [16] but the precise nature of the phenotype remains unclear. More recently, the treatment of patients with omalizumab has been suggested to alter the phenotype of basophils but the underlying mechanisms for the change in phenotype are unclear. Two changes in protein expression during treatment lead to different conclusions [17]. For example, relative decreases in expression of the beta subunit of FcεRI suggest that the basophils in treated patients behave as if IL-3 levels have been decreased. In contrast, syk expression increases in treated patients and increase in syk expression have been associated with exposure to IL-3.

To work towards an understanding of these various phenotypic changes, a more global perspective on the phenotype of basophils was needed and effort was turned to expression profiling to help. These studies began with a general assessment of the mRNA profile of peripheral blood basophils and progressed to assessing the effects of several well known cytokines on the basophil phenotype to provide a context for future comparative studies of basophil phenotypes. There are two cytokines known to have significant functional effects on the basophil, IL-3 and IL-33 as well as cytokines such as IL-5 and NGF that likely share similarity to IL-3. IL-3 is a starting point for basophil studies because of its importance in the development, maturation and maintenance of basophils, as demonstrated in both humans and mice [18–23]. A limited study from our pilot efforts has described a useful IL-3 signature for human basophils [24] but previous studies have demonstrated 3 time frames for the effects of IL-3 [25–29] and differential dependence of some outcomes on the concentration of IL-3 [30]. Each of these characteristics was examined for the entire transcriptome. Finally, although there is a previous report of the effects of IgE-mediated stimulation on the basophil transcriptome [31], this mode of stimulation was re-evaluated in the context of the results for cytokine exposure and because more recent studies have suggested phenotypic changes resulting from chronic aggregation [17,32]. The primary goal of these studies was to determine if there were unique changes in the mRNA profile with different stimuli that could be used to identify the origins of a particular basophil phenotype and to determine the limitations of this approach.

## Methods

### Materials, Buffers and Antibodies

The following were purchased: PIPES, bovine serum albumin (BSA), EGTA, EDTA, D-glucose, NaF, Na_3_VO_4_, 2-ME (2-mercaptoethanol); RPMI 1640 containing 25 mM HEPES and L-glutamine (BioWhittaker, Walkersville, MD); Percoll (Pharmacia, Piscataway, NJ); Tris(hydroxymethyl)-aminomethane, Tween-20 (Bio-Rad,Hercules, CA); anti-CD25 alpha subunit and anti-CD25 beta subunit (CD122) (AbD Serotec, Raleigh, NC); anti-TRPM2 (Genetex, Irvine, Ca); anti-maxi-K channel, (Abcam, Cambridge, UK); anti-FosB (Cell Signaling, Beverly, MA), anti-Egr-3 (Santa Cruz Antibodies, Santa Cruz, CA), anti-HSP90beta (Millipore, Temcula, CA), anti-Orai1 and anti-STIM1 (Proteintech Group, Inc. Chicago, Il.), HRP-conjugated sheep anti-mouse Ig Ab (Amersham Life Science, Arlington Heights, IL). Mouse anti-human IgE Ab (6061P) (Hybridoma Farms, MD). PIPES-albumin-glucose (PAG) buffer consisted of 25 mM PIPES, 110 mM NaCl, 5 mM KCl, 0.1% glucose, and 0.003% HSA. PAGCM was PAG supplemented with 1 mM CaCl_2_ and 1 mM MgCl_2_. Countercurrent elutriation and labeling with antibodies for flow cytometry was conducted in PAG containing 0.25% BSA in place of 0.003% HSA (elutriation buffer). ESB is Novex electrophoresis sample buffer containing 5% 2-mercaptoethanol. Buffers were prepared with RNase and DNase-free reagents where possible.

### Basophil purification

For most of these experiments basophils were purified from leukopheresis packs. They were purified to near homogeneity by sequential application of Percoll gradients, countercurrent elutriation and negative selection using the basophil purification kit (Stem Cell Technologies, Vancouver, BC) and columns from Miltenyi Biotec (Aubum, CA) [33]. The average purity of these basophils by alcian blue staining [34] was 99%. Starting viability of these cells was typically >97%.

The procedure to isolate basophils by the above technique require approximately 2 hours on the blood separator and 5–6 hours at the bench. At the bench, with the exception of the two Percoll gradients used to separate cells, the cells were maintained at 4°C. When basophils were purified from selected donors, the protocol omitted the elutriation step but included enrichment by a two-step Percoll gradient and negative selection as above. Basophil purities averaged 98%. This procedure typically requires 3–4 hours and with the exception of the Percoll separation step, the cells were maintained at 4°C. An Excel spreadsheet enumerating the specific purity, viability and function of the various preparations will be included on the websites (see below) containing the data that will accompany this manuscript.

### Subjects & Ethics

Leukapheresis donors are approved by protocols obtained by the leukapheresis center and the cells used for purification of basophils are considered a residual waste product. The protocol for obtaining blood from the 16 subjects that were examined in this study was approved by the Johns Hopkins IRB and the donors provided a written approval of a consent approved by the same IRB. Written consents are saved as part of the standard record keeping for IRB-approved studies. Four phenotypes were examined; “releaser”, “non-releaser”, atopic and non-atopic (the releaser/non-releasers were also considered non-atopic). Based on historical studies of the non-releaser phenotype [35], the classification of non-releaser was kept strict; release stimulated by a range of anti-IgE Ab concentrations (6061P monoclonal anti-IgE Ab) was not statistically different from unstimulated release (<1%). Releasers were chosen to release greater than 60% with the same antibody. For the pilot screening purposes of this study, atopic subjects were chosen solely on the basis of a history of allergies that included a previous diagnosis by a physician. The category of non-atopics was based on self-reported histories of no known allergies. All sampling was done in a 2 month period in November and December, i.e., outside the normal seasonal allergy exposure time frame for the Baltimore area.

### Reaction conditions

Stimulation or culturing of purified basophils was typically in culture medium consisting of RPMI-1640 supplemented with 1 mM calcium chloride, 8 μg/ml gentamycin and 0.03% RNase free BSA. Culture periods were 2 hours to 3 days. In most cases, a portion of the cells were placed in culture for 15 minutes, then recovered to extract mRNA; this was called the day 0 sample. After culture, recovery and viability were measured with erythrocin B staining. If there were sufficient cells, whole cell lysates were also prepared for Western blotting of proteins. All samples were also processed for mRNA. The early series of experiments used the HighPure RNA Isolation Kit (Roche) but for approximately 70% of the experiments, RNA was isolated with miRNeasy extraction kit (Qiagen) (this was chosen in order to also provide samples for miRNA analysis). Subsequent analysis of the results showed no general differences (average intensities and variation) in the dataset generated from the mRNA obtained by each method. But, closer inspection of the detailed results showed some differences between the methods. Since we also noted that it is not possible to compare profiles obtained on different microarray slides that also included were developed with different service laboratory reagent preparations, the different mRNA preparation methodologies did not pose an impediment in these mostly paired studies. Total RNA was determined both by the RiboGreen RNA Quantitation Kit (Molecular Probes, Invitrogen) and by the Agilent PicoSeries RNA analysis which was also used to determine the RNA quality (RIN, RNA Integrity Number). Most commonly, 250 ng of RNA were submitted to the service core for analysis although there was some variation in the early studies. Assessments of RNA integrity (RIN) generally showed values above 8.0 when the methodology did not also isolate miRNA. But the nature of RIN algorithm causes RIN numbers to be 1–2 points lower when the miRNeasy kit was used for isolation. The first half of the studies were done when the Illumina human Ref 8v3 microarray was in use by the service lab which subsequently switched to Illumina human HT12v4 microarray, which was used in the remaining half of the experiments. S1 Table (a spreadsheet that is included on the two websites noted below) summarizes the conditions of each experiment and characteristics of the basophil preparations.

### Western blots

Pelleted cells were lysed in 20–80 μl of hot ESB, the tube placed in boiling water for 5 minutes and the samples stored at -80°C until electrophoresis. Samples were run in a 15 well 8% tris-glycine gel with molecular weight markers in the first and last lanes. The nitrocellulose was blotted with specific antibodies. We have previously established that p85α is a good lane-loading control antibody [3,36,37] because its expression is unmodified during a wide variety of experimental designs and because it is easily detected with few cells. Multiple film exposures were made in order to optimize linearity, the films were digitized and the band intensities determined by NIH Image.

### Flow cytometry

All analysis was performed on a BD FACSCalibur flow cytometer. Since these experiments were preformed with highly purified basophils, no specific gating antibodies were required. Generally, binding profiles for specific antibodies to a target were compared to the same concentration of an antibody of the same species isotype. Day-to-day instrument variability was monitored using CaliBRITE APC calibration beads (BD Biosciences).

### Data Preparation

The Illumina platform produces a spreadsheet with two columns of numbers for each sample, the readout intensity of a spot on the array and software assessment of the likelihood that the spot is positive. All relevant data are available in the paper, its Supporting Information files and in the Gene Expression Omnibus (accession # GSE64664). Additional data, including an in-house designed application for exploring the ultimate raw data set, are available via 'http://www.basophil.net' and 'http://162.129.217.250/basophilMicroarrays'. The website includes a variety of files one of which provides a detailed description of the analysis methodology. These additional files (tables) are referenced in the manuscript as S1, S2, S3, S4 and S5 Tables.

This study is accompanied by the dissemination of an application that can be used to explore the entire basophil dataset as one series (joining HuRef8 and HT12v4 datasets). The application’s help file will describe in detail the calculations used to analyze the data. The websites noted above will provide a variety of large files. There will be a primary folder/directory containing the application to analyze the data, the main raw Illumina data file for use by the provided application, an application Help manual that describes in detail the algorithms used to analyze the Illumina data and how to use the application, two folders/directories containing useful ancillary files for the application, an Excel spreadsheet describing important details about each experiment that formed the basis of the results presented in this manuscript and a folder/directory containing a variety of large tables or results in spreadsheet form that are labeled S1, S2, S3, S4 and S5 Tables.

### Normalization of microarray results between conditions

Briefly, for analysis, the likelihood-of-positivity value (provided by the Illumina scanner software, Bead Studio or Genonme Studio) was used to determine if the spot should be analyzed; a value <0.015 was the threshold to declare positivity. After inclusion, the data from each column of numbers was normalized by comparing column average intensities for all positive transcripts. The averaging was refined by one of two methods; moment normalization which eliminates the highest intensities so as not to skew the average, or median average where the default is to determine the median intensity. Normalization was done within each set of comparisons rather than first normalizing the entire data set. Because most of the samples contained the same total RNA, normalization effects were modest. Most of the data presented does not subtract the background intensity. In this way, even samples that could be justifiably considered zero intensity can be used in the denominator of ratios. The ratios are blunted to some extent by this approach, particularly when a transcript transitions from not being present to being markedly present. When this is important to consider, it will be mentioned in the results.

### Statistical significance of the changes

Two methods were used to determine thresholds for significant change. The simplest was to calculate the standard deviation for the entire array, i.e., the ratio of two conditions is obtained for each transcript, the data log-transformed, the standard deviation of the ratios of all positive spots determined, and the algorithm iterated once more after removing high and low data (first moment). The resulting global standard deviation was corrected for multiple samples (the Bonferroni correction) to obtain upper and lower thresholds that would be considered statistically significant. This provided the most conservative estimate of thresholds for positivity. When there were replicate experiments, the average standard deviation of replicates was calculated and used to determine upper and lower thresholds for statistically significant changes. This calculation provided the least conservative estimate of thresholds for positivity. To provide an algorithmic estimate that was of intermediate between Bonferroni and replicate thresholds, the Benjamini-Hochberg false discovery rate (FDR) method was calculated.

### Similarity Algorithm

The relative sparseness in the number of replications for a dataset with thousands of features led to a simplified approach for comparison. Similarity between two conditions of stimulation (e.g., between a 24-hr treatment with IL-3 vs. IL-33) was calculated using a simple “distance metric” for the change. For this analysis, the manhattan-distance was calculated for each positive gene [38]. The distance or difference metric between two conditions is the absolute value of the difference between the logarithm of the change ratio for a gene under the two conditions of stimulation. This calculation is also described in greater detail in the help manual for the application provided online. For example, the gene ANXA1 (gene ID 301) decreases 0.39 fold with IL-33 and 0.35 fold with IL-3. The distance metric for this gene alone was 0.047 (|log(0.39)-log(0.37)|, close to zero, where the vertical bars denote absolute value). To determine a threshold difference that could be considered meaningful (i.e., two changes that are not the same), a similar calculation was done except that individual results for replicate conditions were each compared to the average distance metric for all replicate conditions for the same gene (this is analogous to calculating standard deviation). The IL-33 distance metric for individual replicates versus their respective average equals 0.282, an average across all genes analyzed. Therefore, the threshold for similarity was ≤0.564 (2 x 0.282) and the change in the ANAX1 transcript for the IL-33 and IL-3 conditions would be considered similar (0.047 << 0.564) and therefore not a unique transcript for either condition. In contrast, THBS1 (gene ID 7057) changed 3.7 fold with IL-3 and 17 fold with IL-33. The distance metric is, therefore, 0.662, which is outside the threshold for similarity (0.662 > 0. 564) and was therefore considered unique to IL-33. This assessment was applied for each other known stimulus (IL-3, no IL-3, IL-33, anti-IgE Ab, FMLP) and for a gene transcript to be considered unique, it needed to be unique in each comparison. This criterion narrows the number of unique gene transcripts considerably.

Once a set of unique transcripts was chosen, it could be used as a signature of the stimulus. One final variation of the similarity index adjusts the calculation for the possibility that an overall signature set may be composed of transcripts that show mostly large vs. small changes. The similarity index can show a much greater range under these conditions. This effect could be normalized by dividing the similarity index by the average fold change in the signature set (as an analogy to standard deviation in frequency statistics, this is a version of coefficient of variation) but this addition to the calculation was ultimately not found to be necessary.

## Results

### IL-3 induced changes in the transcriptome

To perform these experiments, basophils were purified to near homogeneity, mRNA extracted and analyzed on the Illumina HuRef8 or HTv412 platform. As noted previously [24], at rest, peripheral blood basophils express 8000–11000 transcripts that register as positive according to the Illumina algorithm to detect positives.

We have previously published a useful IL-3 signature for basophils that focused on transcripts that both increase and decrease with or without IL-3 exposure for 18–24 hours. This signature of an IL-3 effect was called the SIL3-51T (**S**ignature of **IL3** using **51 T**ranscripts) and the transcripts that composed this signature had the property of reversing their response when in the presence or absence of IL-3, suggesting that the typical peripheral blood basophil sits in a state somewhere between the two extreme phenotypes. In the published description of how to use this signature [24], the ratio (gene-by-gene of the 51 transcripts) of two conditions is calculated and compared to the signature of treating cells with or without IL-3 for 24 hours. If there is a relative absence of IL-3 (or some equivalent functional effect), a comparison with the ‘noIL3’ signature will generate a positive correlation and comparison with the ‘withIL3’ signature, a negative correlation. The slope for each of the two correlations is an indication of the relative strength of the effect (see below). This report will use this signature to help in the analysis of the remaining results.

The IL-3 response is considerably broader than the 51 transcripts used for the published signature and many transcripts don’t show the reciprocal response characteristic of the transcripts chosen for the published signature. Taking advantage of the paired nature of these experiments allows for a threshold of positivity based on the Benjamini-Hochberg false discovery rate (FDR) threshold leads to a choice for significance at changes greater 3.3 fold (in either direction). The S1 appendix provides greater detail on the general and specific nature of the basophil response to IL-3 (also see S2 Table).

To provide a useful tool for exploring the effects of cytokines and other stimuli, basophils needed to be cultured and there are well known consequences of *in vitro* culture without IL-3, notably apoptosis, although it is slower process in basophils compared to other polymorphonuclear leukocytes [27,29]. For the current studies, a gene signature was developed for what is being called “culture effects”. For this study there are two forms to this signature. Using results from basophils cultured with (10 ng/ml) or without IL-3 (n = 4 experiments), changes in transcripts that occurred regardless of the presence of IL-3 were compiled. This resulted in a list of 181 genes that change regardless of how the cells are handled. The second form of this signature includes genes that change in culture without IL-3. Further information about the selection of these genes is included in the S1 appendix. These two lists were useful as a first pass for screening the changes that occur with all stimuli but a more graded algorithm was also used to finalize the search for unique changes with other stimuli (see below). The fact that there is a ‘cultureEffect’ is interesting because it suggests that *in vitro* studies have a built-in bias but also that once it is possible to study cells that have not needed extensive *ex vivo* preparation that we may come to understand the conditions that define the *in vivo* state.

While the potential relevance of the changes to basophil biology induced by IL-3 is of interest (and is covered more extensively in the supporting information appendix (S1 Appendix), the primary goal of the study was to determine if there are signatures that could be used for analysis of “natural” basophil phenotypes. To this end, there were several issues that were of interest when considering the IL-3 response:

1. IL-3 effects occur on 3 time frames, the fastest of which (2–5 minutes) is likely to be essentially a regulation of post-translational influences [25,39–41]. However, there are differences in the cellular response between 1 and 3 days and this prompts an exploration using the microarrays.
2. A differential dependence on IL-3 concentration of several described functional outcomes [26,30] leads to questions about whether there are clusters of transcripts that significantly differ in their dependence on the concentration of IL-3.
3. IL-3Rα shares a beta subunit with IL-5 and GM-CSF but expression of these receptors appears considerably lower in basophils and functional studies [27] suggest that the functional consequences of IL-3 vs. IL-5 might be distinct. Therefore a comparison between IL-5 and IL-3 was made.
4. NGF appears to share some properties with IL-3 [27] although like IL-5, the functional outcomes to NGF exposure are modest. A comparison of NGF and IL-3 was made.

### Extended treatment with IL-3

Changes in the basophil phenotype induced by IL-3 occur on several time scales, including a time scale of days. Therefore, basophils were also cultured for 3 days with IL-3. As noted previously, a SIL3-51T signature analysis shows that the 51-gene subset behaved similarly between 1 and 3 days.

Non-paired comparisons were made for day 1 and day 3 and these were validated in a couple of experiments directly comparing day 1 with day 3 in the same cells. Since there are functional differences between day 1 and day 3, the focus of the analysis was on changes that distinguished these two time points. Not surprisingly, several patterns could be observed. Approximately 50% of the changes observed for one day of treatment were relatively stable, approximately 40% were transient, with the remaining 10% either showing considerably greater change by day 3 or even reversing direction. Of these remaining 10%, there were only 35 that were different enough to be notable in both paired and non-paired experiments; Table A1 in the S1 appendix shows a curated list of significant changes after removing cultureEffect genes. Some of the functionally relevant transient changes were notable. For example, CD32 (both FcγRIIa and FcγRIIb) transcripts increased at Day 1 but were near starting levels after 3 days. It is possible that these patterns could be used to distinguish a recent exposure to IL-3 vs. more chronic exposure and the transcripts listed in bold were included in the general signature analysis filter discussed below.

### Concentration-dependence of the IL-3 Response

**Fig 1:**
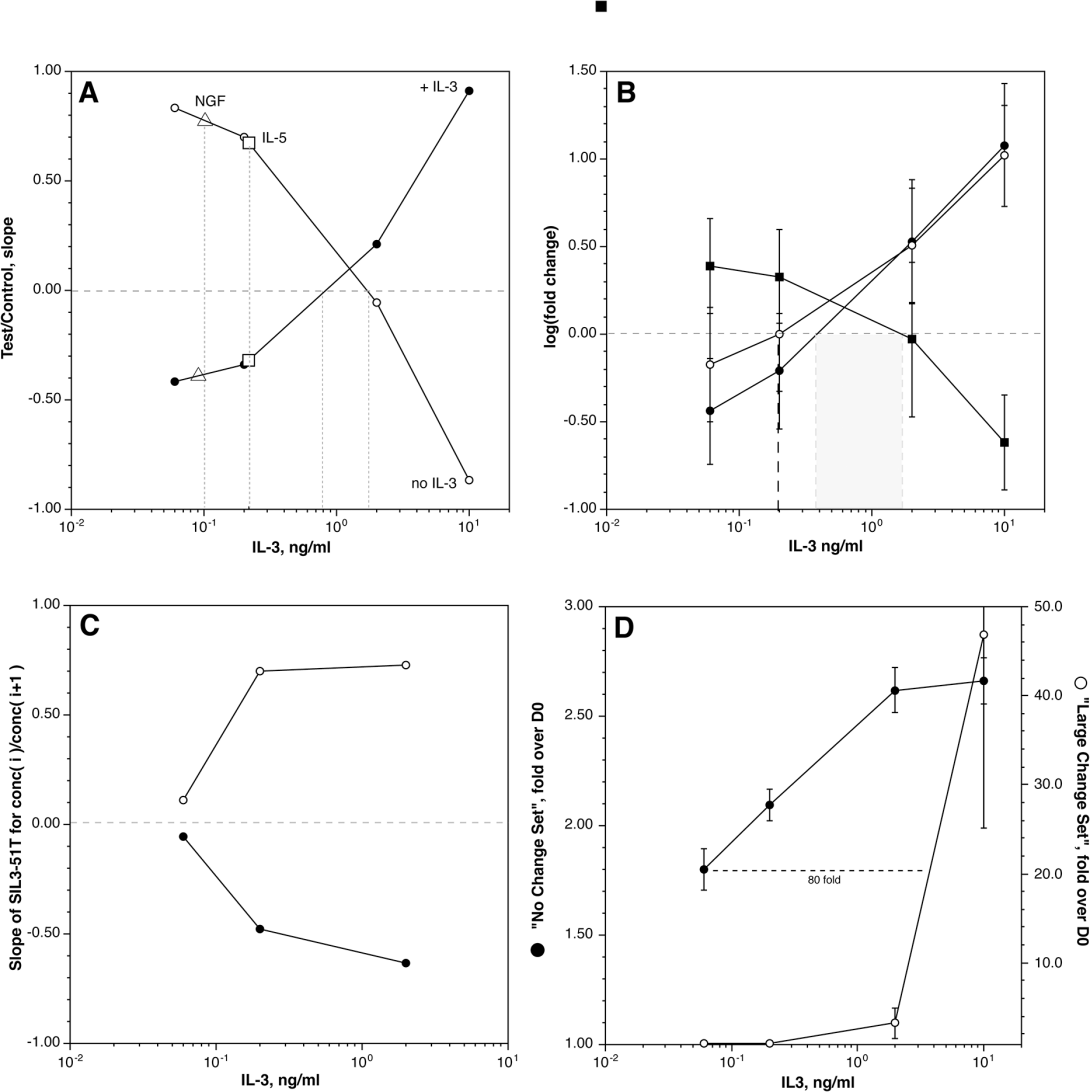
Concentration-dependence of the transcriptome response to IL-3. Panel A: Slope of SIL3-51T gene set for a 24 hour culture at the concentrations shown. The data points for each of the concentrations were derived from different numbers of experiments (n = 2 for 0.06 ng/ml, and n = 4 for 0.2, 2.0 and 10.0 ng/ml) and the plots represent composites of the results from different experiments. The lines represent the comparison with the +IL-3 (●) dataset or the noIL3 dataset (○). The square symbols represent the slopes for an IL-5 at 10 ng/ml exposure (relative to the SIL3-51T test set) and open triangles, the slope for NGF at 10 ng/ml). Panel B: Fold changes in three groups of transcripts. Two groups were derived from SIL3-51T, those that increased with IL-3 (●, n = 34) and those that decreased with IL-3 (■, n = 17). The third group (○) are transcripts that increase greater than 5 fold at the 10 ng/ml IL-3 concentration. Panel C: value of the SIL3-51T relationship between concentrations of IL-3. The ordinate is the slope of the SIL3-51T relationship for IL-3 concentration(i) vs. concentration(i+1) where (i) is a chosen concentration and (i+1) the next higher concentration, for example, 0.06 ng/ml vs 0.2 ng/ml (or 0.2 vs. 2.0, 2.0 vs. 10). The two lines are the slopes for the noIL3 relationship (○) and the +IL3 relationship (●). Panel D: Concentration dependence for two subsets of transcripts chosen with a hierarchical clustering algorithm (see text), those that show similar changes at 2 vs. 10 ng/ml (●) and those that show significant differences at the two concentrations (○).

There are indications that there is considerable variability in the concentrations of IL-3 required to induce different phenotypic effects. We were interested in several related questions that required an analysis of the IL-3 dose response curve. Basophils were stimulated with IL-3 at 10, 2, 0.2, and 0.06 ng/ml IL-3 for 24 hours (although not always in the same experiment due to limitations in basophil number). The first question asked of the results was about the character of SIL3-51T across the dose response curve (this signature set was developed from the two extremes of IL-3, none vs. 10 ng/ml). There are two ways to analyze these results using SIL3-51T. The first uses the slope of the two regression fits that are part of the SIL3-51T analysis (see above); Fig 1A shows that 2 calculated slopes invert at a concentration between 1 and 2 ng/ml (when comparing 24 hours with the respective IL-3 concentration to day 0). Fig 1B shows that a more traditional calculation of fold change for the two subsets of genes (those that increase with IL-3 vs. those that decrease) within SIL3-51T show a crossing point also at approximately 1 ng/ml. Finally, for comparison, Fig 1B also the dose-response relationship is shown for the 109 genes that change greater than 5 fold at 10 ng/ml IL-3.

A related question is whether 2 different concentrations of IL-3 always show the expected relative relationship when analyzed using SIL3-51T since this signature was developed to detect the relative exposure to IL-3. This question is relevant to future comparisons where, *a priori*, it is not known where on the IL-3 dose response curve the experiment starts. Fig 1C indicates that throughout the range of 0.1 to 10 ng/ml, if one compares one concentration to another, SIL3-51T properly indicates the direction of the difference. For example, if incubation in 0.2 ng/ml were compared to 2 ng/ml, SIL3-51T would show the relative absence of IL-3 (where ratios of the changes at 0.2 ng/ml divided by changes at 1 ng/ml were calculated and used in the standard SIL3-51T analysis).

To analyze the broader response to IL-3, a clustering algorithm was used to discover the range of behavior across the dose-response curve. Briefly, after removing ‘cultureEffect’ transcripts from the analysis and excluding transcripts that changed less than 2 fold (increase or decrease after 24 hours), the *ratio* in of the response at 2 vs. 10 ng/ml was used as a metric for a Ward hierarchical clustering algorithm. Clusters were found where the transcripts show a similar increase or decrease at both 2 and 10 ng/ml (vs. day 0), clusters were found that show an extreme difference between 2 and 10 ng/ml and clusters between these extremes were identified. Fig 1D shows the very different dose response curves for the two extreme cluster groups. The cluster that shows very little difference between 2 and 10 ng/ml is composed of 80 transcripts (from approximately 1418 transcripts that change 2 fold or greater) from which 27 show an increase with IL-3 exposure (the rest showing a decrease). The cluster that shows only a response at 10 ng/ml is composed of only 7 transcripts (5 of which increase with 10 ng/ml IL-3, shown in Fig 1B). But the average fold change for the cluster with a low EC50 is only 2.5 fold while the average fold change for the cluster that depends on high IL-3, is nearly 50 fold. Within these two clusters, the EC50 was related to the starting expression level; the high IL-3 dependent cluster showing starting expression levels that are near zero and the low IL-3-dependent cluster showing initial expression levels near the average for basophils. But this relationship between starting expression level and the EC50 for change did not hold for the entire 1418 transcripts that change at least 2-fold. The results might be used to distinguish between exposures to mid and high IL-3 concentrations (see below). For example, if changes in granzyme B expression were to be observed *in vivo*, one might conclude that the cells had been exposed to high concentrations of IL-3.

### Response to IL-5

**Fig 2:**
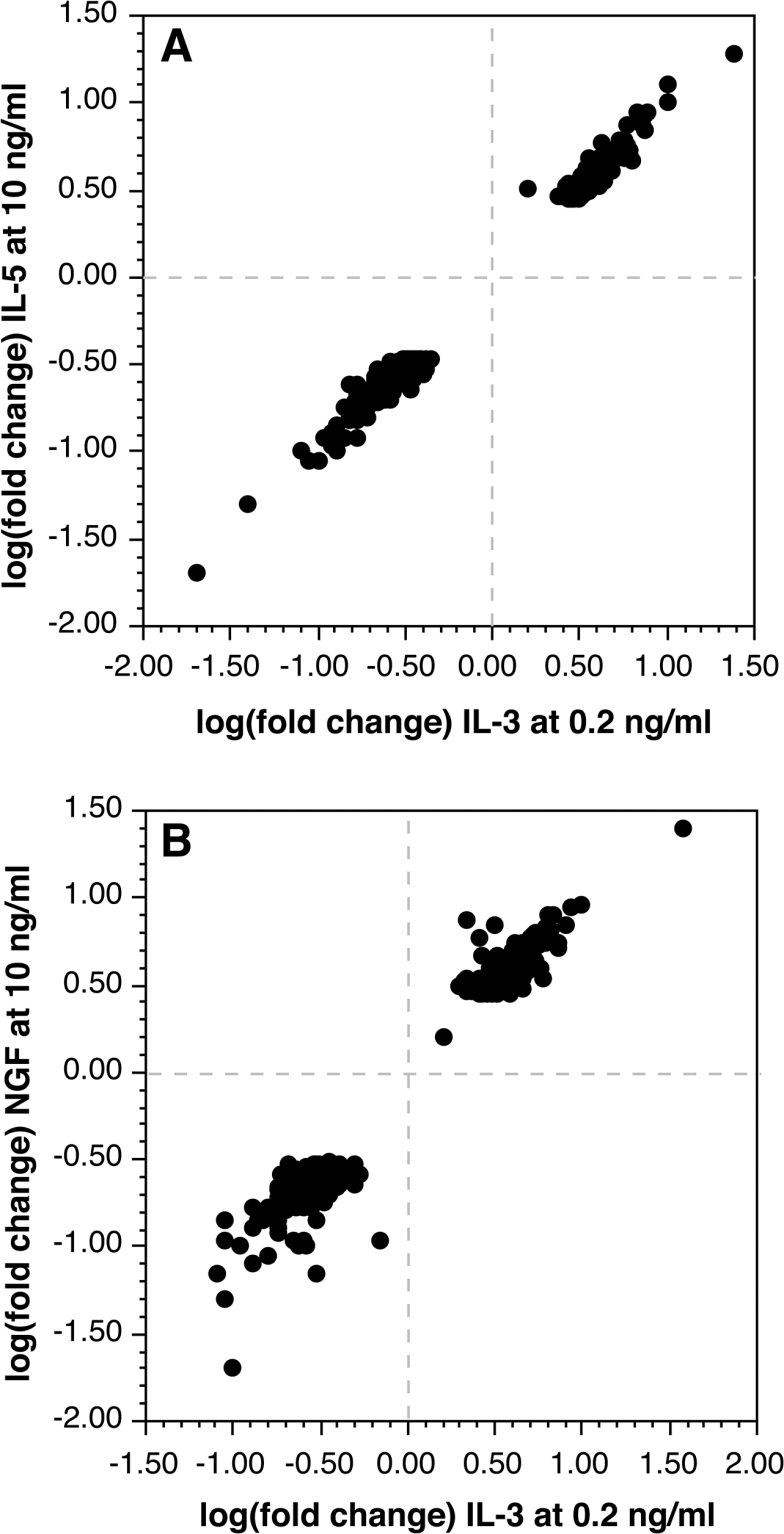
Relationship between changes in transcripts responding to IL-5 or NGF and IL-3. Panel A: IL-5 at 10 ng/ml vs. IL-3 at 0.2 ng/ml. Panel B: NGF at 10 ng/ml vs. IL-3 at 0.2 ng/ml.

The presence of IL-5Rα and the shared IL-3β subunit suggests that basophils may simply have a redundant or vestigial regulation through this receptor. We and others have noted that IL-5Rα appears to be less well expressed than IL-3Rα (with the caveat that antibodies for the two receptors are not typically cross-calibrated) [27,42] and we have observed weaker functional consequences to IL-5 exposure than observed with IL-3. Basophils were treated with 10 ng/ml IL-5 in parallel with an IL-3 dose response curve study. As a first level of analysis, the SIL3-51T signature was examined and as shown in Fig 1A, the response to IL-5 shows a response consistent with somewhat less than 0.2 ng/ml of IL-3. After excluding cultureEffect transcripts, the changes observed with IL-5 are very nearly the same as observed for 0.2 ng/ml of IL-3. Fig 2A shows the tight correlation between changes induced by IL-3 and IL-5. No outliers were observed. For one experiment, 30 ng/ml of IL-5 was compared to 10 ng/ml of IL-5 and there was no meaningful difference suggesting that the results with 10 ng/ml of IL-5 were as significant as could be expected.

### Response to NGF

Previous functional studies noted that NGF induces changes in function that are similar to IL-5 and low concentrations of IL-3 [27,43]. But since this receptor is distinct from IL-3 and IL-5, basophils were examined for a potentially unique signature in the response to 10 ng/ml NGF for 24 hours. The SIL3-51T analysis places NGF in the same region as IL-5, behaving like 50–100 pg/ml of IL-3 (see Fig 1A). Like IL-5, there were no significant outlier changes in response to NGF that would suggest a unique signature. Fig 2B shows the tight correlation in response to NGF at 10 ng/ml vs. and IL-3 at 0.2 ng/ml. The results in Fig 2B are relative to D0 for either IL-3 or NGF but a comparison can be made between D1 IL-3 at 0.2 ng/ml and D1 NGF at 10 ng/ml and the average difference for 382 transcripts (which exclude cultureEffect changes) was 0.96 fold.

### IL-2 induced changes following up-regulation of IL2R by IL-3

Early experiments demonstrated that IL-3 induced large changes in IL-2R (11, 36 and 2.3 fold for alpha, beta and gamma, respectively). Changes in the protein were also noted and discussed in the text of the S1 Appendix and shown in Fig A2 in the S1 Appendix. The appearance of the IL-2 receptor on IL-3 treated basophils led to testing whether IL-2 could mediate interesting changes in the basophil transcriptome. Basophils were first incubated with 10 ng/ml IL-3 for 24 hours after which 10 ng/ml IL-2 was added to the cultures for a second 24 hour period. For comparative purposes, the control was the 2 days with IL-3 alone. In this analysis, the ‘cultureEffect’ filter was used (the noIL3 list was not included since both samples used IL-3). The remaining list of transcripts were only those found for IL-3, or in a direct comparison of IL-2 + IL-3 vs IL-3, no difference was observed.

### Changes in miRNA with IL-3

Five preparations of basophils were examined for resting levels of miRNAs. Two experiments also tested for changes in response to IL-3 for 3 days. In resting cells, there were 160 out of 920 possible miRNAs tested that were considered positive. S3 Table (see methods) lists these miRNAs in descending order of presence. A comparison of D0 and D3 treatment with 10 ng/ml IL-3 showed only modest changes in 15 miRNAs; these are also listed in S3 Table. Unlike the quantitative robustness of mRNA microarrays (see the S1 Appendix), miRNA microarrays are less quantitative, changes less than 5 fold on the miRNA microarray do not generally repeat with qPCR. However, changes larger than 10 fold do appear to hold true when using qPCR (data not shown). In this context, the changes due to IL-3 exposure were minimal, with only one possible change of 10 fold for hsa-miR-21*.

### IL-33 induced changes

IL-33 induced a change in profile that was shared with and differed from IL-3 in substantial ways. First, the SIL3-51T signature indicated a response that was consistent with the absence of IL-3. Interestingly, this analysis revealed that 4 of the transcripts that are part of SIL3-51T behave in a way not consistent with the absence of IL-3 and this observation allows a refinement of the SIL3-51T signature set to exclude these 4 transcripts to further focus the signature on IL-3-dependent changes (a refinement to be incorporated in the combined signature tool below).

Treatment with IL-33 generated a list of ca. 1100 transcripts using a positive threshold based on replicate SD, 75 that used the Benjamini threshold of ±5.85 fold and only 39 using a Bonferroni threshold of ±7.85 fold. But curating the lists by removing cultureEffect transcripts and culture without IL-3 transcripts (to be consistent with the SIL3-51T results), there were only 32 and 10 transcripts remaining from the FDR and Bonferroni lists. Working with the FDR-generated list of 32 transcripts, 22 were unique to IL-33 and 10 changed to a similar degree with IL-3 (the website includes a spreadsheet with these 32 gene transcripts) but this list is further curated using the similarity algorithm below. Further details concerning IL-33 can be found in the S1 Appendix.

### Changes with IgE-mediated stimulation

**Fig 3:**
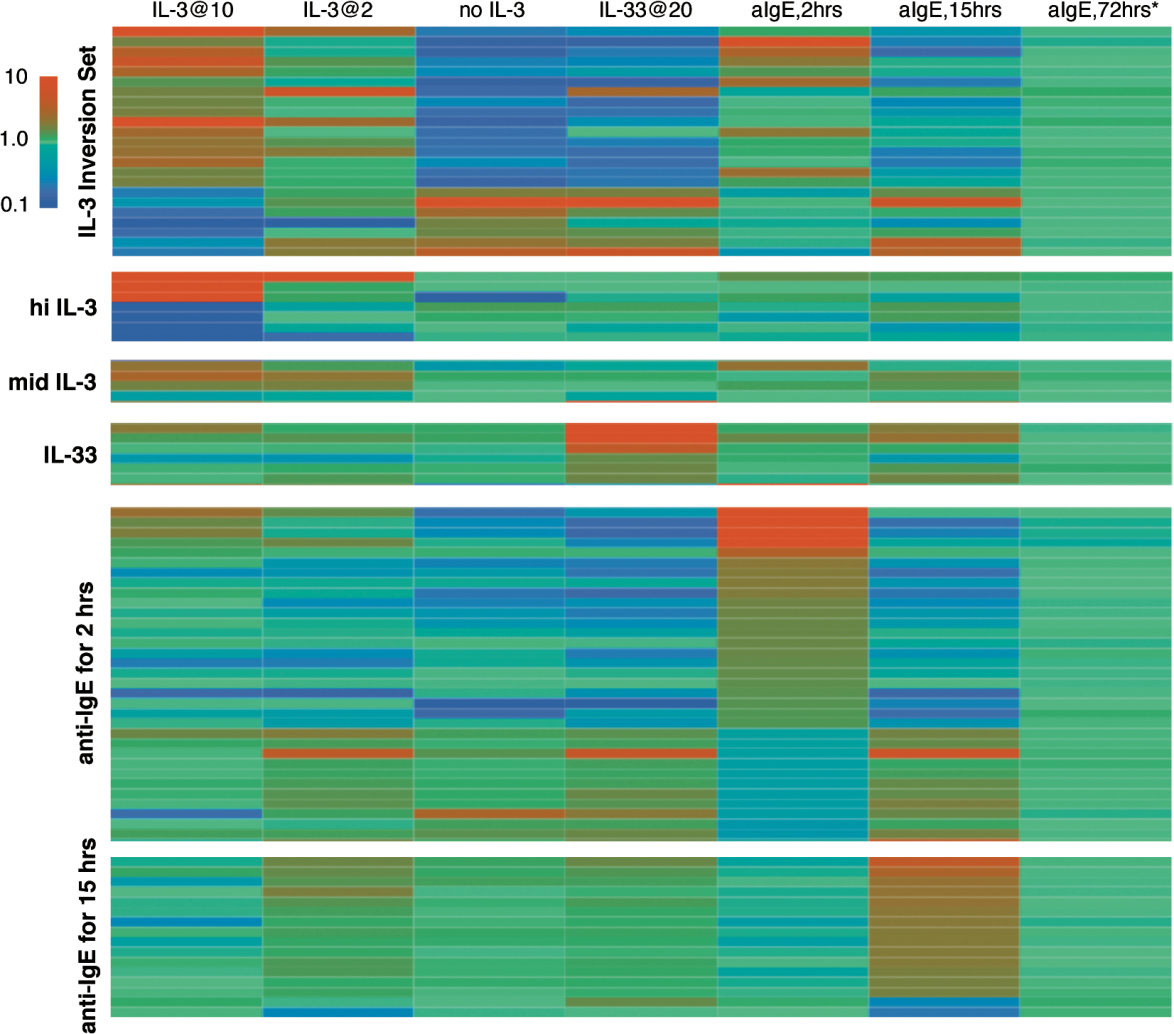
Heatmaps of selected genes to be considered as signatures for stimulation by IL-3, IL-33 and anti-IgE Ab. Seven conditions are presented across the horizontal and 6 potential signature sets along the vertical. There are 24, 7, 4, 6, 34 and 16 genes in the 6 signature sets (see Table A3 in the S1 appendix for a textual listing). The IL-3 conditions include 10 and 2 ng/ml IL-3 for 24 hrs and no IL-3 for 24 hrs. IL-33 at 20 ng/ml for 24 hours and anti-IgE Ab at its optimal concentration of 0.5 μg/ml for 2, 15 and 72 hrs are also shown. The 72 hour incubation for anti-IgE includes IL-3 at 10 ng/ml and the comparison is between IL-3 alone for 72 hours vs. IL-3 plus anti-IgE Ab for 72 hours. All other comparisons are between the stimulation condition and freshly isolated cells (Day 0). The insert shows that the gradient of colors progress from blue for down-regulation of expression to green for no change and red for up-regulation of expression in the range of 0.1 to 10 fold. Values below 0.1 or above 10.0 are blue and red respectively. The signature set labeled ‘IL-3 inversion set’ refers to the genes whose expression behave in opposite directions in the presence or absence of IL-3 and represent a subset of SIL-51T. The set labeled ‘hi IL-3’ are genes that are changed only at 10 ng/ml IL-3 while the set labeled ‘mid IL-3’ are selected from genes that change to a similar extent in both 2 and 10 ng/ml IL-3. As noted in the text, there a 2 potential sets for stimulation with anti-IgE Ab, reflecting differences between 2 and 15 hours of stimulation.

There is one published accounting of the changes induced in the transcriptome in basophils stimulated through the IgE receptor [31]. But we were interested in determining if there are long lasting signatures that can identify the history of a circulating basophil, so basophils were studied using stimulation with anti-IgE Ab over 3 time frames, 2, 15 and 72 hours. The longer incubation required the use of IL-3 so that a comparison at 3 days was between IL-3 alone (for 3 days) or IL-3 + anti-IgE Ab. The 2 and 15 hour incubations did not include IL-3. Based on the one published study [31], the cells stimulated for 2 hours were centrifuged after 30 minutes to remove the supernatant, restored in media plus the same concentration of anti-IgE for the additional 1.5 hours (this was reported to remove feedback changes related to initial secretory events). As noted in our report on the SIL3-51T signature, 2 hours of stimulation with anti-IgE results in a weak signature of the presence of IL-3 (see below, this can also be seen in the heatmap shown in Fig 3). At two hours, there were 128 changes that exceeded the replicate threshold of 2.09 fold and which excluded cultureEffect genes (which notably includes IL-4 because expression of IL-4 transcripts decreases about 2 fold in simple culture; with stimulation, it increases 2.7 fold at the 2 hour time point but from previous studies, the peak is known to occur at approximately 45 minutes). This list is reduced to 34 relatively unique changes that will be discussed further below. With the exception of IL-4, many of the genes on the list were not known to be relevant to basophil biology. None of these changes were evident after 15 hours or 3 days. However, after 15 hours of stimulation a different set of genes became apparent that were unchanged at 2 hours. An initial list of 35 changes that passed through the cultureEffect filter, was further refined (as described below) to a list of 16 (also see the heatmap in Fig 3). None of these genes were different after 3 days. Simply stated, stimulation with anti-IgE for 3 days generated no differences that exceeded Bonferroni adjusted thresholds and only 6 (AMICA, CCL2, CCL4L1, DHR59, HBB, HIST1H4C) that exceeded a threshold defined by the average measured standard deviation between replicates of positive genes (<0.58, > 1.72). Relative to the IL-3-induced changes at 3 days, the changes induced by anti-IgE for 3 days more closely resembled the background “noise” of simple replicate measurements of resting cells (see below and Fig 3).

### Response to FMLP

Basophils respond to several 7-transmembrane, GTP-binding protein linked receptors of which FMLP produces the most marked changes in function. In a pilot experiment, basophils were stimulated with 100 nM FMLP for 15 hours in the absence of IL-3. As with all other non-IL3 stimuli, there was a SIL3-51T signature consistent with the absence of IL-3 and consistent with this behavior were hundreds of changes due to culture. The Benjamini FDR could not be readily calculated for n = 2 and the Bonferroni threshold of 8.88 fold resulted in 10 changes, of which only 2 remained after removing cultureEffect genes. No changes were unique to FMLP (relative to IL-3, noIL-3, IL-33 and anti-IgE Ab, see below). A single experiment of a 2 hour challenge showed only 19 significant changes, none of which were unique to FMLP, so further studies were not pursued.

### Signatures

To develop possible signatures for each of the cytokines examined, a similarity algorithm was employed. In place of using the qualitative yes-no process used to exclude “cultureEffect” changes or changes associated with apoptosis (or no IL3), the similarity algorithm uses the quantitative results of the microarray. The methods section and online information describe this algorithm. This process eliminates cultureEffect and no-IL3 genes just as the manually curated exclusion lists did but has the virtue of including the entire dataset for all conditions in the decision process. For example, when this approach is used to search for unique changes caused by IL-33, 4–7 genes remain that are distinct for IL-33 (a variable number because this approach allows for some adjustment in the threshold for inclusion in a unique set). For the anti-IgE response at 2 hours, 34 genes stand out, for the anti-IgE response at 15 hours, 16 genes stand out. While FMLP shows 28 changes that don’t intersect with IL-3 or noIL-3, adding anti-IgE and IL-33 into the comparative filter reduces the unique selection to 0. Therefore, there were no clearly distinct changes associated with FMLP. The analysis of the IL-3 dose response sensitivity adds some genes that distinguish between high dose IL-3 and mid-dose IL-3. Note that IL-5, NGF, or IL-2 cannot be distinguished on the basis of unique signatures from the 4 conditions above or from an IL-3 or no IL-3 signature alone. Finally, part of a general signature analysis includes the previously published SIL3-51T that allows one to test for the relative presence or absence of IL-3. Table A3 in the S1 Appendix lists the transcripts part of the signature set.

**Fig 4:**
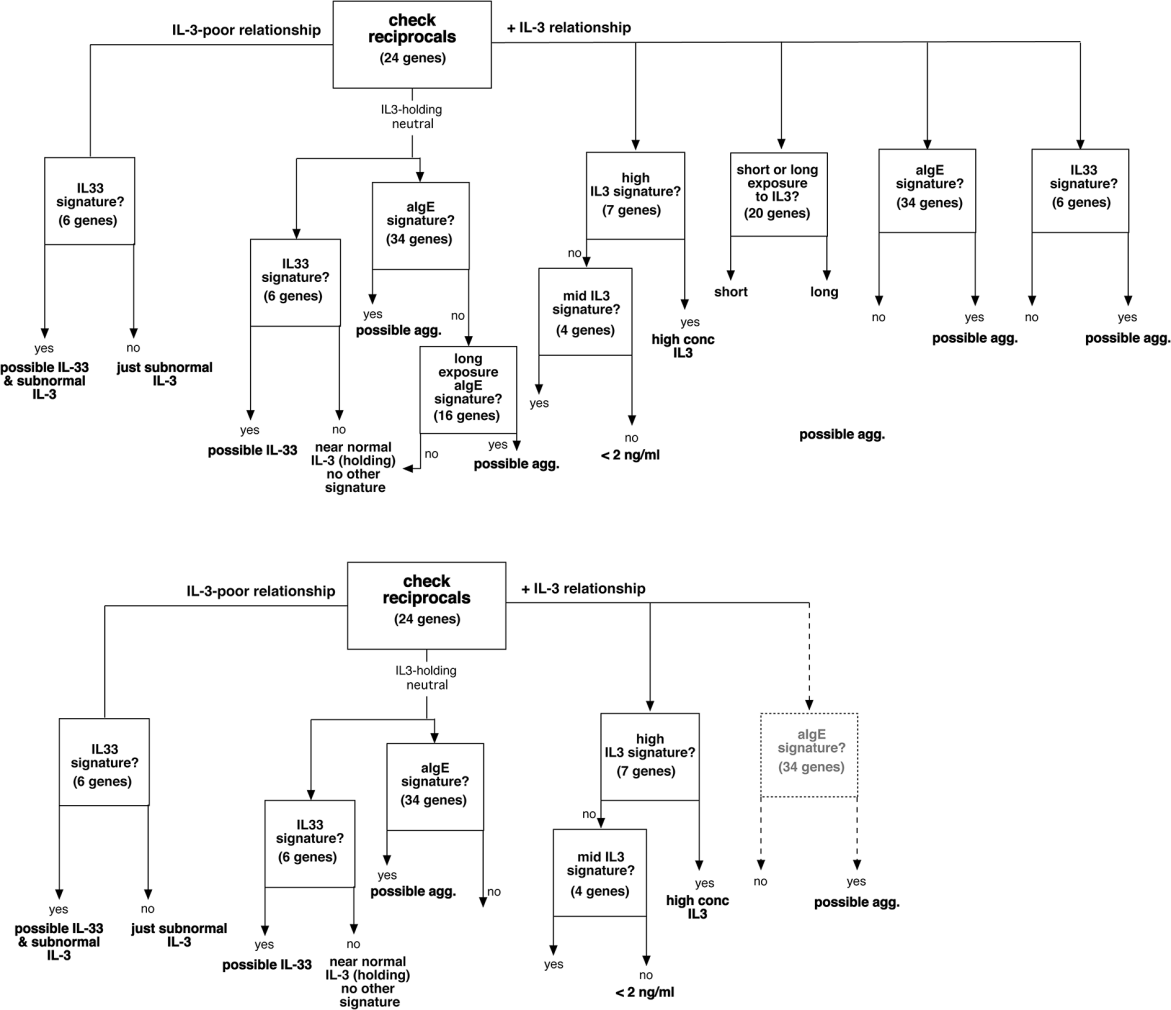
Design of a signature algorithm that incorporates the results for the various stimuli. The top half shows the algorithm that was explored using the existing information and the bottom half the algorithm pruned for those elements that were found to be consistent with the results.

Fig 4 shows how a signature similarity matching could be used to detect the presence of stimulation by IL-3, poor IL-3, long or short IL-3, IL-33 and IgE-mediated stimulation. The transcripts used for each of the similarity tests are listed in Table A2 of the S1 Appendix. Fig 3 shows a heatmap of the changes associated with each of the signature sets for 7 conditions of stimulation. If a weak signature of IL-3 were detected, it would not be possible to distinguish it from high concentrations of IL-5 or NGF. The flow diagram in the top half of the figure was the initial logic based on potential signature genes. The logic reflects the recognition that IL-3 (or no IL-3) dominates the behavior of basophils in these *in vitro* cultures (see next paragraph) and is therefore placed as the first analysis in the logical flow, after which, other signatures are analyzed. It includes the possibility that IL-33 and IgE-mediated stimulation could be detected in the presence of a detectable IL-3 exposure and included the possibility that a 15-hour IgE-mediated stimulation could be detected. However, after exploring the various conditions in the entire dataset, it became apparent that pruning of the flow diagram was necessary. As noted above, IL-3 exposure suppresses the IL-33 signature. For this reason the anti-IgE signature, in the presence of a positive IL-3 signature (right side of the flow diagram) is included with qualification. It is apparent that in culture, IgE-mediated stimulation generates a weak IL-3 signature and the anti-IgE-signature was derived from these conditions and is therefore valid. But whether a basophil that is exposed to IL-3 for a significant period of time prior to stimulation through FcεRI shows the same aIgE signature or the signature is modified/blunted as observed with IL-33 (see S1 Appendix) is unclear without further testing. In addition, while a comparison of short and long term exposure was a possibility if the starting point was freshly isolated leukocytes, it became apparent that these signatures were less reliable if some prior exposure to IL-3 was the starting point (at least *in vitro*). Finally, the 15-hour anti-IgE Ab signature appeared to show similarity to a variety of other conditions, such as poor IL-3, high IL-5 or high NGF. The bottom half of Fig 4 shows the logical flow that the data supports.

**Fig 5:**
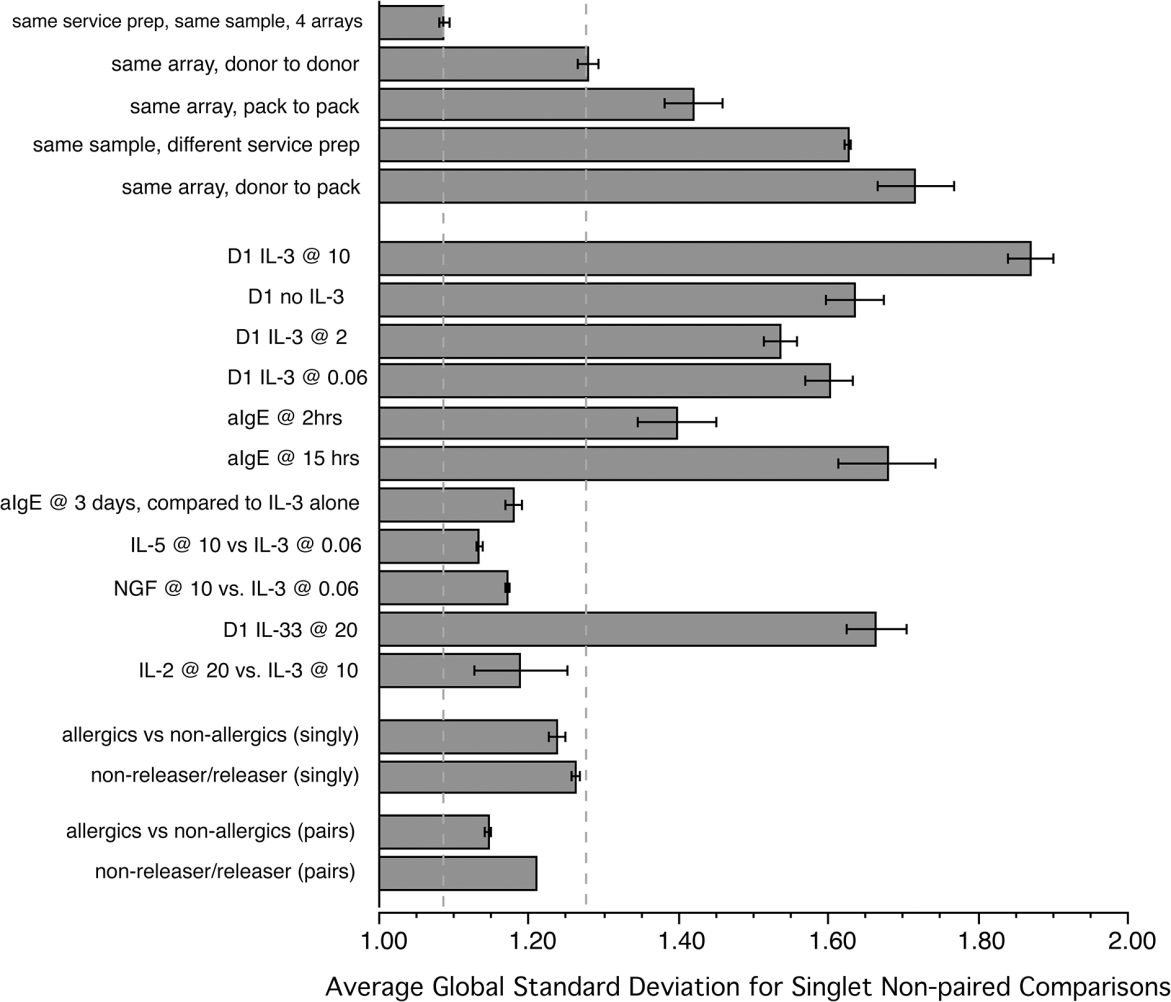
Use of global standard deviation as a metric of the relative magnitude of changes for a variety of comparisons.

In order to obtain a sense of the stability of the microarray profiling, this study initially generated some samples for comparison that were analyzed on the microarrays in various ways. As has been noted in other microarray studies, the optimal design of comparative studies is to run samples 1) on the same microarray slide or 2) run samples on multiple microarray slides but using the same reagent preparations for amplification (in the figure and in the spreadsheet describing the experiments (S1 Table), this is referred to as same service preparation) and finally, at a minimum, the same RNA isolation techniques and the same type of microarray. Fig 5 summarizes an analysis that used the global standard deviation of ratios for the entire microarray results as a metric of various comparisons (i.e., if many large changes occurred during stimulation, the global SD would be larger than if no changes occurred). The samples used in these calculations varied but included the same leukapheresis preparations, different leukapheresis preparations, and basophils isolated by standard venipuncture. In this later case, the donors were chosen from several categories; non-atopic subjects, atopic subjects, and subjects whose basophils had previously been classified as non-releasers or releasers. Some of the comparisons were part of earlier pilot experiments and are not explicitly part of the list of experiments listed in S1 Table. Because some of these comparisons were necessarily non-paired, all the comparisons were done with a non-paired analysis. In addition, in some cases the global standard deviation could only be calculated for non-paired singlets, so this was used throughout even when multiple samples were present so that the global standard deviation did not reflect the smoothing of variation due to replication. As expected, comparing the same sample on the same microarray slide generated the least amount of general variation. There was more variation when comparing donor to donor on the same array and comparison of standard venipuncture samples to leukapheresis samples increased the variation to a point that suggested this was not a reliable comparison. Not shown is that the basophil isolation methods must be the same if a non-paired comparison is made and that results cannot be compared across different service preparations of reagents (although different slides with the same service preparation of reagents was nearly as good as same slide comparisons).

With these guidelines, Fig 5 shows with two dotted lines a range of variation that might be considered expected for donor-to-donor comparisons. In the middle section of the figure is shown the global variation with various types of stimulation and it is clear that IL-3 or its absence for a one day incubation creates the largest amount of variation. In this group are some comparisons that fall within the expected range for little difference. With these results providing a context, it can be seen that there are no differences in the 3 categories of venipuncture-derived samples. Six samples each from non-atopic and atopic subjects (who were sampled outside of the seasonal allergy periods but not perennial allergen seasons) were run on the same HuRef12 microarray slide and 2 samples each from releasers and non-releasers were run on a separate microarray slide. Comparing releasers to non-releasers or atopics to non-atopics resulted in global variation that was within the range expected for little or no differences. Use of the simple global standard deviation as a metric is limited since specific but low frequency differences would be hidden in the global calculation. Using the general signature tool outlined in Fig 3, there were no changes consistent with differential exposure of the any of the groups to any of the stimuli examined in this study. Furthermore, a simple search for any changes between the non-atopic and atopic groups reveals only 5 transcripts with a difference between 2.0–3.0 fold. Indeed, the differences between the groups were similar to comparing a sample to itself.

## Discussion

This survey provides a first look at the regulation of basophil mRNA expression by several cytokines and secretagogues and the results provide a potential tool for examining the status of basophils with respect to exposures *in vivo*. One potential limitation of the approach is that simple isolation of basophils from circulation induces a series of changes that are significant. It is not surprising that apoptotic changes would be one aspect of the basophil response to isolation but it is apparent that some changes occur regardless of the presence or absence of IL-3, which is generally known to prevent apoptosis in basophils, at least for a period of 24–48 hours. Indeed, relative to other polymorphonuclear leukocytes, basophils are relatively resistant to apoptosis [27,29] (note the viability and recovery statistics in S1 Table, 95% viability and 83% recovery), so changes that occur with or without IL-3 may not only represent initiation of apoptosis. For convenience, these non-IL-3 dependent changes have been termed cultureEffect changes but their precise origin and function are unknown. It is also unknown whether these changes can mask the effects of other non-IL-3 stimuli. This would be difficult to examine because only exposure *in vivo* could provide the necessary information and a controlled exposure *in vivo* is not likely to be done. With this concern in mind, the results suggest that of the stimuli examined, IL-3, or its lack, dominates the behavior of basophils and the behavior of the transcriptome. There were 700–900 gene transcripts that change greater than a threshold derived from replicate variation (without adjustment for multiple tests) with or without IL-3. The Benjamini-Hochberg FDR thresholds resulted in 229 and 100 changes respectively for 10 ng/ml IL-3 and no IL-3. In contrast with these numbers, use of an FDR threshold generated 0 positives for IgE- and FMLP-mediated at 2 hours and 75 for 20 ng/ml IL-33 from which, only 5 are not excluded because they are also expected from the cultureEffect and the absence of IL-3. Relaxing the threshold to the average replicate limits for positivity yields 129 changes for IgE-mediated stimulation but even here, the changes are relatively modest (3–5 fold range with a few exceptions). This general picture of influence of the various stimuli is recapitulated in the analysis of variation shown in Fig 5. The domination by IL-3 (or its absence) suggests a logical flow to evaluating a basophil phenotype based on its mRNA profile. This logic is shown in Fig 3 and the primary node in the flow of logic is an evaluation of the relative presence of IL-3 using a subset of the SIL-51T signature presented previously. In the current version, 24 transcripts define the reciprocal signature and if it shows a relative absence of IL-3, the ability to analyze the profile for other influences is restricted to IL-33. If the response is neutral, then the response could be analyzed for an influence by IgE-mediated stimulation or IL-33. Finally, if there is a signature of relative IL-3 exposure, 4 signatures of the IL-3 response could be assessed. If the IL-3 exposure is from high concentrations of IL-3, this might be distinguished from mid-level IL-3 exposure. Use of SIL-51T (or newer SIL3-24T indicates that between D1 and later times, there a slight tendency towards IL-3 depletion in culture. But there is also the potential to distinguish exposures that are in the 24 hour range vs 72 hour range because some transcripts show large swings between these time points. It also appears that the signature of long IL-3 exposure only works well if the starting condition is a “resting” basophil, i.e., if the first measurement were made longer than 12–24 hours after exposure to IL-3 then these length-of-time signatures don’t seem to apply. This may restrict the use of this particular signature. However, at this time there is no way to confirm whether these signatures would faithfully identify these characteristics *in vivo*. It is also apparent from these *in vitro* studies that there is no unique signature for IL-2, IL-5 and NGF and that IL-5 and NGF look similar to low concentrations of IL-3. In addition, using the 15 hr signature for anti-IgE Ab under a variety of other conditions, e.g., comparing day 1 and day 2 IL-3, suggests that this signature will be difficult to interpret. Therefore, while some of these signatures may be useful to suggest possible influences, the most conservative interpretation of the results is that exposure to IL-3 or its lack is likely to be detectable, while short term exposure to IgE-mediated stimulation or longer exposure to IL-33 only have a modest chance of being detectable.

The primary goal of the study was to determine if unique signatures could be found for known ways to stimulate basophils but these studies are also the most complete exploration of the basophil transcriptome to date. There are many interesting observations about basophil biology that follow from these measurements. Some interesting examples are explored in the S1 Appendix and some of the observations made in the early development of this dataset have helped define new areas of basophil biology.

Although the purpose of this study was not to determine the precise relationship between the changes in mRNA and the proteome of the basophil, it is apparent that the two outcomes are not always concordant. In general, we found that the Illumina microarray generated very consistent results that matched information obtained by qPCR, i.e., the rough changes detected by the microarray appear validated. However, the translation of these changes to protein expression was, as a general statement, blunted. More often than not, changes in mRNA on the order of less than 3–10 fold did not result in a similar change in the protein. There were some interesting exceptions but it is clear that this survey can only be used as a tool to identify signatures that may have utility in identifying candidate protein changes or utility for expression profiling.

Of the cytokines examined, only IL-33 induced changes both significant in effect and distinct from IL-3. This may be expected since studies by Pecaric-Petkovic et al [44] indicate that even acute changes induced by IL-33 (despite a very low level of ST2 expression) are mediated through an NFkB pathway, unlike the pathways initiated by acute exposure to IL-3 [27]. From the standpoint of discovering unique signatures to identify the potential causation of basophil phenotypes, the response to IL-33 is perhaps not unique enough to design an assay. Nearly 60% of the changes follow those induced by IL-3 as well as being proportional to changes induced by IL-3. This suggests a shared group of transcriptional regulators for both cytokines. Although it appears that approximately 15% of the changes induced by IL-33 are potentially useful since the direction of change is opposite to IL-3, further testing with the similarity algorithm (with comparisons to other stimuli) removes these from the list of possibly unique transcripts. Likewise, 30% of the changes do not occur with IL-3 (or its absence) but a close examination of the utility of these differences suggests only 6 that might clearly be distinguished from background and exposure to IL-3. Nevertheless, 4–7 transcripts might be useful as a signature of IL-33 exposure.

It was somewhat surprising that chronic exposure to an aggregating stimulus did not result in any additional permanent changes in mRNA transcripts. It was apparent that the fairly modest changes induced by FcεRI aggregation were short-lived, requiring less than 1 day to return to the nominal state. Because the basophil cannot be cultured for more than 18 hours without progression into apoptosis, the 3-day cultures with anti-IgE antibody required IL-3 (note that the IL-3 generated by IgE-mediated stimulation alone is not sufficient to prevent apoptosis over many days [45]) and it could be that the marked changes in transcript expression induced by IL-3 swamped any effects due to chronic aggregation. But even a 15 hour incubation without IL-3 showed that the changes observed in the first few hours were transient. Nevertheless, there were some changes in transcripts that were found to distinguish IgE-mediated signaling from IL-3-induced signaling. There is increasing evidence that changes observed *in vitro* may be blunted relative to changes that can occur *in vivo*. For example, synthesis of FcεRIα is markedly blunted *in vitro*, perhaps by 20 fold [46,47]. Likewise, synthesis of FcRβ is nearly non-existent *in vitro* [48], even with IL-3 present, but is clearly present *in vivo*. It is possible that aggregation-induced changes are more marked or longer-lived *in vivo* and therefore more detectable but this will need to be determined.

In summary, it appears possible to distinguish the mRNA profile of basophils exposed to IL-3, IL-33 and IgE-mediated stimulation but stimulation with IL-2, IL-5, NGF or FMLP does not generate a unique signature and likely does not generate any change that is anymore remarkable that the progression of the cell into apoptosis or its adaptation to culture conditions.

## Supporting Information

S1 Appendix Text of the appendix describing the changes induced by IL-3, IL-33 and anti-IgE Ab. Fig A1: IL-3 dose response curves for the expression of CD32a (●) or CD32b (○). The mRNA expression results are derived from the microarray results. The grey lines show protein expression results derived from a previous study [12] and these results are plotted as a fraction of the maximum increase observed at 10 ng/ml of IL-3. Fig A2: Flow cytometry for cell surface IL-2alpha on purified basophils. Panel A shows the profile for day 0 basophils, light gray line is the isotype control antibody and the darker line, the anti-CD25 antibody; panel B is for Day 2 IL-3 treated basophils. Panel C is derived from another experiment, day 0 profiles, and Panel D, the day 3 profiles. Table A1: Differences between changes in expression following IL-3 on Day 1 (n = 6) vs. Day 3 (n = 5). Text in bold highlights notable differences. Table A2: Fidelity of the microarray changes as assessed by either qPCR or Western blotting for the related protein. In some cases, the comparison was made between the microarray and qPCR while in other cases, the comparison was between the microarray and protein changes assessed by either Western blots or flow cytometry. * measured at 45 minutes, ** measured after 18 hours incubation. None of the comparisons are matched samples, i.e., these are unpaired comparisons that are reflective of the general changes observed. Table A3: Listing of the unique genes used for the combined signature analysis shown in Fig 4. There are several groups that test for the presence of IL-3, high or low concentrations of IL-3, short or long exposure to IL-3, exposure to IL-33 or IgE-mediated stimulation. In some of the table cells labeled ‘Ratios’, there are two numbers. When present, the genes are used in two similarity determinations or, in the case of the IL-3 reciprocal set, used for two correlations. For the genes labeled ‘24 reciprocals’, the first ratio is the training set determined change in the absence of IL-3 and the second number, the change in the presence of IL-3. For the genes labeled ‘short vs. long IL-3’, the first number is the ratio after 24 hours of exposure and the second number is the ratio after 72 hours.
(ZIP)

**S1 Table. Synopsis of 26 experiments used in the analysis.** Where measured, the viability and recovery of basophils after culture is noted. The starting basophil purity is noted and where relevant, the percent histamine with an optimal concentration of anti-IgE Ab is noted. The column designated as CHIP# shows which Illumina slide (numbered to distinguish one slide from another) an experiment was run on. The column designated Service # shows which reagent preparation was used to run an experiment. This refers to the practice by the service lab of running a set of experiments on different slides but with the same amplification reagent preparation. It is provided to allow comparisons, where appropriate, that should yield optimal results. Available on http://www.basophil.net and http://162.129.217.250/basophilMicroarrays.

**S2 Table. IL-3 induced changes that exceed the Benjamini-Hochberg FDR of 0.30 to 3.31 fold for IL-3 at 10 ng/ml (n = 11 experiments).** The left-most columns (n = 289) do not exclude cultureEffect genes, the middle set of columns (N = 207) exclude the cultureEffect genes and the rightmost set of columns show the same as the middle but ranked for the magnitude of change. Available on http://www.basophil.net and http://162.129.217.250/basophilMicroarrays.

**S3 Table. Basal expression of miRNA in basophils and the effect of IL-3 on miRNA expression.** The data is unprocessed from the miRNA arrays and not normalized for differences in average intensity. There were 5 experiments for basal levels and in two of these 5, a paired set of data for treatment with IL-3 at 10 ng/ml for 3 days. The average intensity data is provided for the 5 experiments and the average intensities for experiments 4 and 5 for the D0 time point and the D3 IL-3 at 10 ng/ml time point. Available on http://www.basophil.net and http://162.129.217.250/basophilMicroarrays.

**S4 Table. Culture Effect list used to ignore changes that occur regardless of the presence of IL-3.** The list is not exhaustive but curated for consistency across the two Illumina array products used in these studies. Available on http://www.basophil.net and http://162.129.217.250/basophilMicroarrays.

**S5 Table. IL-33 response: selection of 32 transcripts that resulted from the Benjamini FDR threshold filter after subtracting cultureEffect and no IL-3 genes.** The list is sorted for those changes similar to IL-3 vs. those that are not. Available on http://www.basophil.net and http://162.129.217.250/basophilMicroarrays.
