# Supplementary material for: Expression Profiling of Human Basophils: Modulation by Cytokines and Secretagogues
Source: PLoS One. 2015 May 11;10(5):e0126435. doi: 10.1371/journal.pone.0126435 (PMC4427102; doi:10.1371/journal.pone.0126435)
Supplement: S1 Appendix — Fig A1: IL-3 dose response curves for the expression of CD32a (●) or CD32b (○). The mRNA expression results are derived from the microarray results. The grey lines show protein expression results derived from a previous study [12] and these results are plotted as a fraction of the maximum increase observed at 10 ng/ml of IL-3. Fig A2: Flow cytometry for cell surface IL-2alpha on purified basophils. Panel A shows the profile for day 0 basophils, light gray line is the isotype control antibody and the darker line, the anti-CD25 antibody; panel B is for Day 2 IL-3 treated basophils. Panel C is derived from another experiment, day 0 profiles, and Panel D, the day 3 profiles. Table A1: Differences between changes in expression following IL-3 on Day 1 (n = 6) vs. Day 3 (n = 5). Text in bold highlights notable differences. Table A2: Fidelity of the microarray changes as assessed by either qPCR or Western blotting for the related protein. In some cases, the comparison was made between the microarray and qPCR while in other cases, the comparison was between the microarray and protein changes assessed by either Western blots or flow cytometry. * measured at 45 minutes, ** measured after 18 hours incubation. None of the comparisons are matched samples, i.e., these are unpaired comparisons that are reflective of the general changes observed. Table A3: Listing of the unique genes used for the combined signature analysis shown in Fig 4. There are several groups that test for the presence of IL-3, high or low concentrations of IL-3, short or long exposure to IL-3, exposure to IL-33 or IgE-mediated stimulation. In some of the table cells labeled ‘Ratios’, there are two numbers. When present, the genes are used in two similarity determinations or, in the case of the IL-3 reciprocal set, used for two correlations. For the genes labeled ‘24 reciprocals’, the first ratio is the training set determined change in the absence of IL-3 and the second number, the change in the presence of IL [file pone.0126435.s001.zip › Supporting Information Appendix/Supporting Information text.pdf]

Supporting Information Appendix

Selecting the "cultureEffect" genes

A subtlety to the choice of 'cultureEffect' genes when comparing the effects of culture in the presence and absence of IL-3 is that the changes did not need to be identical (which is unlikely) but within several fold of each other when the changes were in the same direction. In addition, the lesser of the changes in the two conditions needed to be greater than 2.0 fold to pass through this algorithmic filter. One example is the FcεRIbeta subunit of the IgE receptor, which decreases 0.04 fold without IL-3 and decreases 0.21 fold in the presence of 10 ng/ml IL-3 (this is an observation that has been reported previously [1] that is re-capitulated in the microarray results). There are 129 transcripts that decrease under both conditions and 52 that rise resulting in a 'cultureEffect' list of 181 transcripts (this list is Table S4 on the websites). In some instances, it is also useful to add to this list the transcripts that only change in the absence of IL-3 because, *a priori*, some stimuli may produce the equivalent of this absence. Using this sub-list as an exclusionary criterion runs the risk of outright excluding changes that are remarkably different from the absence of IL-3 alone. As an example, ITK increases with and without IL-3 but the change without IL-3 is only 2.2 fold while it is 25 fold with IL-3. Using the noIL3 list to filter out transcripts might miss some interesting changes not related to the absence of IL-3. Therefore the threshold for inclusion of a transcript on the noIL-3 list was designed to include only transcripts that showed significant changes; 5 fold (in either direction). This added 129 transcripts to the 'cultureEffect' list; the combined list will be called the 'cultureEffect+noIL3' list.

Relationship to Protein Expression

Table A2 summarizes our experience in relating several of the changes for the microarray dataset and either mRNA changes measured by qPCR or Western blotting for protein. Generally, in those instances where qPCR was used to detect changes in mRNA expression, the results agreed reasonably well with values determined using the microarray approach. For example, previous studies demonstrated that FcRβ mRNA expression increases approximately 3-4 fold, a value similar to the 4.8 fold change in these microarray studies [2,3]. Likewise, the increase in syk expression with IL-3 treatment in the microrrays averaged 3.7 fold and in qPCR, averaged 4.0 fold. Parenthetically, FcεRIα is not represented on the HuRef8v3 microarray and given the importance of this protein in basophil biology, the samples used in the microarray were evaluated by qPCR. Treatment with IL-3 did not result in changes in FcεRIα mRNA, an average of 0.89±0.10 fold change. Seven samples were run using the HT12v4 microarray where a probe for FcεRIα is represented and treatment with IL-3 for one

day also resulted in no change (0.98±0.01). Likewise, IgE-mediated changes in IL-4 mRNA 2 hours after stimulation agree with qPCR results (note that the peak response for IL-4 mRNA occurs at 45-60 minutes after stimulation with a peak change of approximately 15 fold) [4,5]. However, the relationship to changes in protein expression are not concordant or predictable. In general, protein expression was always blunted relative to changes in mRNA and often, no changes were observed in the expected protein despite increases or decreases in mRNA. The discordance was also apparent in IL-3 dose response curve results. Fig A1 shows the changes in mRNA for CD32a and CD32b and the figure shows an overlay of surface expression data obtained from a recent study of these receptors by our group. It is clear that the protein and mRNA results don't show the same EC50. But the relationship between protein and mRNA will probably require a more complete analysis of the temporal changes in mRNA at each concentration because it is likely that protein expression is related to the time integral of the mRNA response. While the basis for the discordance needs further study, the CD32 characteristics might be representative of many other changes.

Categories of change

Other than the focus on unique signatures for basophil stimuli, there is interest in the nature of the changes induced by these stimuli. This appendix will examine some of the interesting changes, or lack thereof, that follow stimulation with IL-3, IL-33 and anti-IgE Ab. The various spreadsheets made available online summarize some of the results and provide a starting point for submitting the results into pathways analysis tools such as DAVID.

IL-3 Stimulation

For IL-3, there were hundreds of changes not associated with the cultureEffect genes. There were five functional areas that were of interest given past understanding of the effects of IL-3 on basophil functions, changes in:

1) ion channels that might influence the cytosolic calcium response
2) signal transduction proteins
3) transcription factors
4) surface receptors
5) granule contents

1) It is has been previously shown that >24 hours treatment with IL-3 results in marked changes in the cytosolic calcium response that follows stimulation with a variety of secretagogues [6,7]. The most evident changes occur for stimulation with C5a, which does not, under the conditions of fresh basophil isolation, induce a second phase (the influx phase) calcium response unless cells are treated with IL-3 for >18 hours. But the changes in ion

channel transcripts was modest. The HuRef8v3 array detected a statistically significant (based on replicate errors) presence for CACNG6, CLCN7, CLIC1, KCNJ2, KCNK6, ORAI1, SCNM1, STIM1, TRPC4AP, TRPM6, TRPV2 and did suggest IL-3-induced changes in CLIC1 (1.9 fold), KCNJ2 (2.8 fold), KCNK17 (4.1 fold), ORAI1 (2.3 fold), TRPM2 (6.6 fold, which was not expressed without IL-3), TRPV2 (2.2 fold, which is expressed at moderate levels in resting cells) and KCNMB3 (a maxi-K channel subunit, 1.8 fold). The Illumina HuRef8v3 array did not represent ORAI2 but the HuRef12 array did; the only changes occurred with ORAI1 (2.3 fold). The changes in ORAI1, TRPM2 and the maxi-K channel subunits were examined by qPCR and/or Western blotting. At the protein level, no statistically significant changes were observed in ORAI1; Day1(+IL-3)/Day0 was 1.24±0.11 fold ($p = 0.08$, $n=5$) and Day3(+IL-3)/Day0 was 1.26±0.11 fold ($p = 0.057$, $n=6$). Likewise, at the protein level, there was no difference in the maxi-K subunit proteins. Western blots showed only questionable bands in the proper region for TRPM2 (≈170 kDa) and there were no changes in expression with a 3 day IL-3 treatment (data not shown). Therefore, there were no clear indications for changes in ion channels that would alter the cytosolic calcium response. The Illumina microarray did not completely represent all known ion channel transcripts. For example, the TRP family is rather large, at least 33 members, the Illumina HuRef8 array represented only 11 while the HuRef12 array represented 26.

2) A variety of signaling elements known to be relevant to IgE-mediated signaling in human basophils were of interest. In some cases, the transcripts were either not present or barely present. For example, PLCδ3, PLCδ4, PLCγ1, PKCγ2 and PKCζ transcripts were not present and PLCδ1, SOS2, PLCβ1, PLCβ2, cbl-b, c-cbl, fyn, PKCε, PKCξ were only weakly evident. Protein expression for these genes is easily demonstrated by Western blot. Previous studies demonstrated that basophils express SOS2 and not SOS1 protein [8], so the absence of SOS2 transcripts but highly expressed SOS1 transcripts is puzzling. It is notable that IL-3 treatment did not alter the expression of transcripts for btk, any of the PLC enzymes, SHIP1 or SHIP2 (whose protein changes 3-6 fold [9]), lyn, p85α (subunit of PI3K), CIN85, BOB1 (POU2AF1), any of the PI3 kinase enzymes, cbp/PAG (PAG1), SHP-1, PTEN, or any PKC enzymes. There were changes in LAT (3.1 fold), SOS1 (5.2 fold), and syk (3.7 fold). There were also several interesting transcripts related to general signal transduction, for example, DUSP1, RGS1, DUSP16, IRS2, MAPKAPK3, FBXO6, RASD1, DUSP5, CISH and SLAMF1, several of which are related to MAPK signaling pathways, an area of change that we have previously identified as one of the changes induced by IL-3 [10].

3) There were some notable changes in receptor proteins (IL-2R, CXCR4, CD11a, EBI2, TNFR-SF4, SIGLEC6, IL17RB, GPR56 and CD300LF). The change in IL17RB transcripts raises the possibility for the addition of a receptor for IL-25 on basophils. There was also a 36 fold change in IL-2Rβ, a 3 fold change in IL-2Rα and 1.8 fold change in IL-2Rγ (the changes are actually greater than this calculation indicates because the transcripts for these proteins were not present prior to culture and became present with culture, see methods). There are modest

changes in CRLF2 (the TSLPR, see IL-33 section below) but this transcript shows the low EC50 for IL-3 that characterizes a large group of genes.

The change in IL-2R (CD25) was further explored by flow cytometry, comparing the expression of CD25 on day 0 and day 2 or 3 (based on studies noted below, the change in IL-2Rα and IL-2Rβ were similar after one day of culture with IL-3, so an intermediate culture length was used for some of the flow cytometric studies). The expression of CD25α appears complex. In resting cells there is little expression with the exception that there is sometimes a positive tail to the distribution but the median for the distribution is only marginally different than the isotype control. After culture, the distribution becomes normally distributed and the median is different than the isotype control (Fig A2). The differences between day 0 and day 3 cells varied considerably, averaging a 2.5 fold increase (range 1.5-4.5). The changes in surface CD25β, detected by flow cytometry, were also modest. By Western blot, the changes in CD25α were also modest (0.9 – 5.9 fold). There were no changes in HLA-DR transcripts although there was modest expression of HLA-DRA transcripts and very poor expression of the DR1-5 series.

4) There were several changes in transcription factors. Some notable transcription factor transcripts were IER3, FOS, FOSB, HOXA5, and GFI1 and two chromatin re-organization-related transcripts, NCOR2 and RCBTB2. The changes to these transcripts was large but some of the changes were not strictly a function of IL-3 actions but instead related to isolating or culturing the cells.

5) There were only a few changes to proteins that might be packaged in the basophil granule. As described previously by Tschopp et al. [11], there was a 136 fold change in granzyme B (GZMB). Cathespin L (CTSL1) and eosinophil major basic protein increased 23 and 26 fold respectively.

IgE-mediated stimulation

A similar analysis of the 5 areas of interest examined for IL-3 reveals a very short list of recognizable and understandable changes. There is a short list of cytokines and chemokines (IL-4, IL-3, IL-8, CCL5, CCL3, CCL3L1, CCL3L3 and CCL4L1) consistent with previous studies. There is only one obvious ion channel (KCNJ2) and a few potentially interesting signal transduction elements, LAX1, DUSP5, SOCS1, SOCS2, PTPN7, CISH, SH2D2A, MAPK13, PLCXD1 and SGK. Most of these are first thought of as signal termination molecules that might be considered consistent with a response to restore the activated state of the cell. However, most of the changes are modest and possibly result in only subtle changes in protein if the experience presented in Table A2 is a guide. There are several transcription factors that change, IER3, NFKB1D, EGR1, 2 & 3 although most of these change with other forms of

stimulation and are not qualitatively unique for IgE-mediated stimulation. There are few potentially interesting receptor changes, C5AR1 (complement 5 receptor), NTRK1 (TRKA receptor, see NGF response), and CD69. There are inconsistent changes (2 of the 3 experiments) in CRLF2 but since IL-3 induces modest increases in this transcript even at low concentrations, it is possible this response represents an autocrine feedback response due to the secretion of IL-3 from IgE-mediated stimulation of basophils. The elevation also persists through the first 15 hours. In the last category, there are no obvious changes in granule protein mRNAs, which might be surprising given the degranulation response of the cell.

IL-33 stimulation

The changes induced by IL-33 after removing changes associated with culturing and the absence of IL-3 were very restricted. But a couple were notable for the implied biology. On this list were CRLF2 (a subunit of the TSLP receptor) and THBS (thrombosponin)(both showing approx. 20-fold increases). While both anti-IgE Ab and IL-3 induce a modest increase in CRLF2, the change with IL-33 is considerably greater. Given the current interest in the role of TSLP in the atopy, this may be an interesting observation except that the TSLP receptor works in tandem with CD127 (IL7R) and the changes to this component are modest following exposure to IL-33 (≈2 fold).

These results for IL-33 can also be viewed from the perspective of whether changes induced by IL-3 occur with IL-33. Working from a list of 469 changes greater than 2.5 fold induced by IL-3 (and excluding cultureEffect transcripts), for 339 of these changes, there was no change induced by IL-33 and for a subset of these, 31 change more than 10 fold with IL-3 treatment but do not change following IL-33. The remaining 130 transcripts show modest change with IL-33, 17 in the opposite direction of IL-3, with rest following IL-3. Only for those changes that increase following either IL-3 or IL-33 is there a correlation. An interesting example is the AQP9 (aquaporin 9) increases approximately 20 fold with both IL-33 and IL-3. If the transcriptional program of AQP9 were known, there might be a clue to the shared aspects of regulation by IL-3 and IL-33.

These 24 hour tests demonstrate that while there were very few unique changes induced by IL-33, it did initiate a mixed program of changes that included many genes that changed with IL-3, but sometimes in an opposite direction. But IL-3 up-regulates the receptor of IL-33, ST2, on basophils so a couple of experiments examined whether the incubation with IL-3 changes the response to IL-33. Changes that had been shown to be specific for IL-33 were found to be completely blunted by the prior IL-3 treatment; (for 33 genes the log fold change for IL-33 stimulation without prior IL3 treatment = 0.652±.247 and after IL-3 treatment, 0.05±0.11, an average 4.5 fold change blunted to 1.13 fold). In a subset of the transcripts that normally change in a direction opposite of IL-3, IL-3 also dominated the response, reversing the

response to IL-33 (or from the perspective of IL-3, IL-33 did not alter the normal progression of events mediated by IL-3). Looking at only the transcripts that changed in opposite directions for IL-3 & IL-33, from D1 to D2, changes in the presence of IL-3 alone averaged 0.36±0.03 (log-fold change) and with IL-33 included, = 0.37±0.04 (log-fold change).

Appendix Figure and Table Legends

Fig A1: IL-3 dose response curves for the expression of CD32a (●) or CD32b (○). The mRNA expression results are derived from the microarray results. The grey lines show protein expression results derived from a previous study [12] and these results are plotted as a fraction of the maximum increase observed at 10 ng/ml of IL-3.

Fig A2: Flow cytometry for cell surface IL-2alpha on purified basophils. Panel A shows the profile for day 0 basophils, light gray line is the isotype control antibody and the darker line, the anti-CD25α antibody; panel B is for Day 2 IL-3 treated basophils. Panel C is derived from another experiment, day 0 profiles, and Panel D, the day 3 profiles.

Table A1: Differences between changes in expression following IL-3 on Day 1 (n=6) vs. Day 3 (n=4). Text in bold highlights notable differences.

Table A2: Fidelity of the microarray changes as assessed by either qPCR or Western blotting for the related protein. In some cases, the comparison was made between the microarray and qPCR while in other cases, the comparison was between the microarray and protein changes assessed by either Western blots or flow cytometry. * measured at 45 minutes, ** measured after 18 hours incubation. None of the comparisons are matched samples, i.e., these are unpaired comparisons that are reflective of the general changes observed.

Table A3: Listing of the unique genes used for the combined signature analysis shown in figure 4. There are several groups that test for the presence of IL-3, high or low concentrations of IL-3, short or long exposure to IL-3, exposure to IL-33 or IgE-mediated stimulation. In some of the table cells labeled 'Ratios', there are two numbers. When present, the genes are used in two similarity determines or, in the case of the IL-3 reciprocal set, used for two correlations. For the genes labeled '24 reciprocals', the first ratio is the training set determined change in the absence of IL-3 and the second number, the change in the presence of IL-3. For the genes labeled 'short vs. long IL-3', the first number is the ratio after 24 hours of exposure and the second number is the ratio after 72 hours.

S1 Table: Synopsis of 26 experiments used in the analysis. Where measured, the viability and recovery of basophils after culture is noted. The starting basophil purity is noted and where relevant, the percent histamine with an optimal concentration of anti-IgE Ab is noted. The column designated as CHIP# shows which Illumina slide (numbered to distinguish one slide

from another) an experiment was run on. The column designated Service # shows which reagent preparation was used to run an experiment. This refers to the practice by the service lab of running a set of experiments on different slides but with the same amplification reagent preparation. It is provided to allow comparisons, where appropriate, that should yield optimal results.

Available on http://www.basophil.net and http://162.129.217.250/basophilMicroarrays.

S2 Table: IL-3 induced changes that exceed the Benjamini-Hochberg FDR of 0.30 to 3.31 fold for IL-3 at 10 ng/ml (n=11 experiments). The left-most columns (n=289) do not exclude cultureEffect genes, the middle set of columns (N=207) exclude the cultureEffect genes and the rightmost set of columns show the same as the middle but ranked for the magnitude of change.

Available on http://www.basophil.net and http://162.129.217.250/basophilMicroarrays.

S3 Table: Basal expression of miRNA in basophils and the effect of IL-3 on miRNA expression. The data is unprocessed from the miRNA arrays and not normalized for differences in average intensity. There were 5 experiments for basal levels and in two of these 5, a paired set of data for treatment with IL-3 at 10 ng/ml for 3 days. The average intensity data is provided for the 5 experiments and the average intensities for experiments 4 and 5 for the D0 time point and the D3 IL-3 at 10 ng/ml time point.

Available on http://www.basophil.net and http://162.129.217.250/basophilMicroarrays.

S4 Table: Culture Effect list used to ignore changes that occur regardless of the presence of IL-3. The list is not exhaustive but curated for consistency across the two Illumina array products used in these studies.

Available on http://www.basophil.net and http://162.129.217.250/basophilMicroarrays.

S5 Table: IL-33 response: selection of 32 transcripts that resulted from the Benjamini FDR threshold filter after subtracting cultureEffect and no IL-3 genes. The list is sorted for those changes similar to IL-3 vs. those that are not.

Available on http://www.basophil.net and http://162.129.217.250/basophilMicroarrays.

References (in Appendix)

1. Saini SS, Klion AD, Holland SM, Hamilton RG, Bochner BS, et al. The relationship between serum IgE and surface levels of FcepsilonR on human leukocytes in various diseases: Correlation of expression with FcepsilonRI on basophils but not on monocytes or eosinophils. J Allergy Clin Immunol 2000;106: 514-520.
2. Miura K, Saini SS, Gauvreau G, MacGlashan DW, Jr. Differences in functional consequences and signal transduction induced by IL-3, IL-5 and NGF in human basophils. J Immunol 2001;167: 2282-2291.
3. Saini S, Richardson JJ, Wofsy C, Lavens-Phillips, Bochner B, et al. Expression and modulation of FceRIa and FceRIb in human blood basophils. J All Clin Immunol 2001;107: 832-841.
4. MacGlashan DW, Jr., White JM, Huang SK, Ono SJ, Schroeder J, et al. Secretion of interleukin-4 from human basophils: The relationship between IL-4 mRNA and protein in resting and stimulated basophils. J Immunol 1994;152: 3006-3016.
5. MacGlashan DW, Jr. Desensitization of IgE-mediated IL-4 release from human basophils. J Leuk Biol 1998;63: 59-67.
6. MacGlashan DW, Jr., Hubbard WC Interleukin-3 alters free arachidonic acid generation in C5a-stimulated human basophils. J Immunol 1993;151: 6358-6369.
7. Miura K, MacGlashan DW, Jr. Dual phase priming by interleukin-3 for leukotriene C4 generation in human basophils. J Immunol 2000;164: 3026-3034.
8. Miura K, Lavens-Phillips S, MacGlashan DW, Jr., Localizing a control region in the pathway to LTC4 secretion following stimulation of human basophils with anti-IgE antibody. J Immunol 2001;167: 7027-7037.
9. MacGlashan DW, Jr. Relationship Between Syk and SHIP Expression and Secretion from Human Basophils in the General Population. J Allergy Clin Immunol 2007;119: 626-633.
10. Vilarino N, Miura K, MacGlashan DW, Jr. Acute IL-3 priming up-regulates the stimulus-induced Raf-1-Mek-Erk cascade independently of IL-3-induced activation of Erk. J Immunol 2005;175: 3006-3014.
11. Tschopp CM, Spiegl N, Didichenko S, Lutmann W, Julius P, et al. Granzyme B, a novel mediator of allergic inflammation: its induction and release in blood basophils and human asthma. Blood 2006;108: 2290-2299.
12. MacGlashan D, Jr., Moore G, Muchhal U Regulation of IgE-mediated signalling in human basophils by CD32b and its role in Syk down-regulation: basic mechanisms in allergic disease. Clin Exp Allergy 2014;44: 713-723.
