# Supplementary material for: Expression Profiling of Human Basophils: Modulation by Cytokines and Secretagogues
Source: PLoS One. 2015 May 11;10(5):e0126435. doi: 10.1371/journal.pone.0126435 (PMC4427102; doi:10.1371/journal.pone.0126435)
Supplement: S1 Appendix — Fig A1: IL-3 dose response curves for the expression of CD32a (●) or CD32b (○). The mRNA expression results are derived from the microarray results. The grey lines show protein expression results derived from a previous study [12] and these results are plotted as a fraction of the maximum increase observed at 10 ng/ml of IL-3. Fig A2: Flow cytometry for cell surface IL-2alpha on purified basophils. Panel A shows the profile for day 0 basophils, light gray line is the isotype control antibody and the darker line, the anti-CD25 antibody; panel B is for Day 2 IL-3 treated basophils. Panel C is derived from another experiment, day 0 profiles, and Panel D, the day 3 profiles. Table A1: Differences between changes in expression following IL-3 on Day 1 (n = 6) vs. Day 3 (n = 5). Text in bold highlights notable differences. Table A2: Fidelity of the microarray changes as assessed by either qPCR or Western blotting for the related protein. In some cases, the comparison was made between the microarray and qPCR while in other cases, the comparison was between the microarray and protein changes assessed by either Western blots or flow cytometry. * measured at 45 minutes, ** measured after 18 hours incubation. None of the comparisons are matched samples, i.e., these are unpaired comparisons that are reflective of the general changes observed. Table A3: Listing of the unique genes used for the combined signature analysis shown in Fig 4. There are several groups that test for the presence of IL-3, high or low concentrations of IL-3, short or long exposure to IL-3, exposure to IL-33 or IgE-mediated stimulation. In some of the table cells labeled ‘Ratios’, there are two numbers. When present, the genes are used in two similarity determinations or, in the case of the IL-3 reciprocal set, used for two correlations. For the genes labeled ‘24 reciprocals’, the first ratio is the training set determined change in the absence of IL-3 and the second number, the change in the presence of IL [file pone.0126435.s001.zip › Supporting Information Appendix/Table A1.pdf]

GeneName	Gene ID	D1/D0	D3/D0
**Transient increases**			
**PRG2**	5553	26	3.6
**MMP1**	4312	7.1	1.4
**FCGR2A**	2212	7.5	1.4
**FCGR2B**	2213	5.3	1.6
ACSS2	55902	5.5	1.1
EDARADD	128178	5.3	1.8
GPR109B	8843	4.9	1.6
KCNJ2	3759	4.1	1.7
**Transient decreases**			
**HIST1H1C**	3006	0.14	1.00
CECR1	51816	0.28	1.50
MYD88	4615	0.19	0.64
NPC2	10577	0.28	0.88
SSH2	85464	0.13	0.54
TOMM7	54543	0.21	0.62
USF2	7392	0.27	0.76
ZNF511	118472	0.43	0.90
**Further change between D1 & D3**			
EMP	2012	5.3	28.0
LGALS1	3956	3.0	15.0
FLJ14123	79899	3.8	14.0
MRPS6	64968	4.3	11.0
NGK7	4818	2.1	8.2
LRP5L	91355	0.4	0.09
LGALS12	85239	0.18	0.1
S100P	6286	0.2	0.04

GeneName	Gene ID	D1/D0	D3/D0
**Rise then fall**			
**DUOXA2**	405753	7.3	0.4
**IL1RL1**	9173	4.1	0.38
PDE4B	5142	2.2	0.21
**Fall then rise**			
**FFAR2**	2867	0.31	3.5
FBP1	2203	0.46	3.1
ID2	3398	0.48	3.3
ITPR3	3710	0.50	2.1
TMC6	11322	0.67	2.7
**Not significant on D1 but significant on D3**			
**DPYSL2**	1808	0.94	7.7
**NKG7**	4818	2.10	8.2
**NEK6**	10783	1.10	5.4
**LAGE3**	8270	0.88	6.5
**NCOR2**	9612	1.10	6.5
PKM2	5315	2.70	6.6
HAVCR2	84868	1.40	5.6
HES6	55502	1.10	3.5
HNRPLL	92906	1.20	3.2
DUSP16	80824	0.48	0.09
GPR65	8477	0.88	0.18
ITGAX	3687	0.89	0.24

Table A1
