# Supplementary material for: Expression Profiling of Human Basophils: Modulation by Cytokines and Secretagogues
Source: PLoS One. 2015 May 11;10(5):e0126435. doi: 10.1371/journal.pone.0126435 (PMC4427102; doi:10.1371/journal.pone.0126435)
Supplement: S1 Appendix — Fig A1: IL-3 dose response curves for the expression of CD32a (●) or CD32b (○). The mRNA expression results are derived from the microarray results. The grey lines show protein expression results derived from a previous study [12] and these results are plotted as a fraction of the maximum increase observed at 10 ng/ml of IL-3. Fig A2: Flow cytometry for cell surface IL-2alpha on purified basophils. Panel A shows the profile for day 0 basophils, light gray line is the isotype control antibody and the darker line, the anti-CD25 antibody; panel B is for Day 2 IL-3 treated basophils. Panel C is derived from another experiment, day 0 profiles, and Panel D, the day 3 profiles. Table A1: Differences between changes in expression following IL-3 on Day 1 (n = 6) vs. Day 3 (n = 5). Text in bold highlights notable differences. Table A2: Fidelity of the microarray changes as assessed by either qPCR or Western blotting for the related protein. In some cases, the comparison was made between the microarray and qPCR while in other cases, the comparison was between the microarray and protein changes assessed by either Western blots or flow cytometry. * measured at 45 minutes, ** measured after 18 hours incubation. None of the comparisons are matched samples, i.e., these are unpaired comparisons that are reflective of the general changes observed. Table A3: Listing of the unique genes used for the combined signature analysis shown in Fig 4. There are several groups that test for the presence of IL-3, high or low concentrations of IL-3, short or long exposure to IL-3, exposure to IL-33 or IgE-mediated stimulation. In some of the table cells labeled ‘Ratios’, there are two numbers. When present, the genes are used in two similarity determinations or, in the case of the IL-3 reciprocal set, used for two correlations. For the genes labeled ‘24 reciprocals’, the first ratio is the training set determined change in the absence of IL-3 and the second number, the change in the presence of IL [file pone.0126435.s001.zip › Supporting Information Appendix/Table A2.pdf]

Table A2

Protein	Condition	Fold-µarray	n	Fold qPCR	n	Fold protein	n
FcεRIbeta	day 3 IL3	4.8	7	3.5-16	5	2.0-30	7
FceRIalpha	day 1 IL3	1.0	7	0.9	8		
syk	day 3 IL3	3.7	7	4.0	7	1.40±0.15	9
Orai 1	day 1 IL3	3.3	2	5.3	2	1.24±0.11	5
	day 3 IL3	2.3	7	5.2	2	1.26±0.11	6
STIM1	day 1 IL3	0.8	2			1.16±0.09	5
	day 3 IL3	1.2	7	1.15	7	1.14±0.14	6
IL-4	2 hr aIgE	3.0	2	5.5*	2		
maxi-K	day 1 IL3	5.0	3			1.14±0.08	2
TRPM2	day 3 IL3	6.2	7			1.15±0.20	2
IL-2Ralpha-flow	day 3 IL3	3.0	7			3.0±1.5	3
IL-2Ralpha-western	day 3 IL3					3.1±1.5	3
IL-2Rbeta-flow	day 3 IL3	79.0	7			1.3	1
HSP90beta	day 1 IL3	2.0	7				
	day 3 IL3	2.9	7			0.86±0.04	2
IRX3 – flow	day 1 IL3	93	11			1.53	2
Egr-3	2 hr aIgE	19.0	2			1.0±0.06**	3
FosB	2 hr aIgE	2.0	2			2.0±0.10**	4
CXCR4	day 1 IL3	0.01	11			0.77±0.03	2
CD62L(SELL)	day 1 IL3	0.02	11			0.53±0.07	2
HOXA5 – flow	day 1 IL3	0.03	11			1.16	1
