# Supplementary material for: Expression Profiling of Human Basophils: Modulation by Cytokines and Secretagogues
Source: PLoS One. 2015 May 11;10(5):e0126435. doi: 10.1371/journal.pone.0126435 (PMC4427102; doi:10.1371/journal.pone.0126435)
Supplement: S1 Appendix — Fig A1: IL-3 dose response curves for the expression of CD32a (●) or CD32b (○). The mRNA expression results are derived from the microarray results. The grey lines show protein expression results derived from a previous study [12] and these results are plotted as a fraction of the maximum increase observed at 10 ng/ml of IL-3. Fig A2: Flow cytometry for cell surface IL-2alpha on purified basophils. Panel A shows the profile for day 0 basophils, light gray line is the isotype control antibody and the darker line, the anti-CD25 antibody; panel B is for Day 2 IL-3 treated basophils. Panel C is derived from another experiment, day 0 profiles, and Panel D, the day 3 profiles. Table A1: Differences between changes in expression following IL-3 on Day 1 (n = 6) vs. Day 3 (n = 5). Text in bold highlights notable differences. Table A2: Fidelity of the microarray changes as assessed by either qPCR or Western blotting for the related protein. In some cases, the comparison was made between the microarray and qPCR while in other cases, the comparison was between the microarray and protein changes assessed by either Western blots or flow cytometry. * measured at 45 minutes, ** measured after 18 hours incubation. None of the comparisons are matched samples, i.e., these are unpaired comparisons that are reflective of the general changes observed. Table A3: Listing of the unique genes used for the combined signature analysis shown in Fig 4. There are several groups that test for the presence of IL-3, high or low concentrations of IL-3, short or long exposure to IL-3, exposure to IL-33 or IgE-mediated stimulation. In some of the table cells labeled ‘Ratios’, there are two numbers. When present, the genes are used in two similarity determinations or, in the case of the IL-3 reciprocal set, used for two correlations. For the genes labeled ‘24 reciprocals’, the first ratio is the training set determined change in the absence of IL-3 and the second number, the change in the presence of IL [file pone.0126435.s001.zip › Supporting Information Appendix/Table A3.pdf]

Gene name	Gene ID	Ratios		Gene Name	Gene ID	Ratios	
CCL23	6368	0.23; 12.0	**24 reciprocals**	THBS1	7057	23	**IL33 set**
CCL3	6348	0.12; 3.9	**for IL3 detection**	CRLF2	64109	18	
CCL5	6352	0.12; 4.1		OLR1	4973	10	
CISH	1154	0.26; 6.7		SOD2	6648	4.2	
DCUN1D3	123879	0.3; 8.5		PTPRC	5788	3.7	
DUSP5	1847	0.11; 2.5		STAG3L1	54441	3	
IL17RB	55540	0.16; 2.9					
FLJ14213	79899	0.16: 4.2		EGR1	1958	20	**34 aIgE(2hr) set**
IFITM1	8519	0.29; 3.4		CCL3L1	6349	14	
LST1	7940	0.18; 3.3		CCL3L3	414062	14	
LTB	4050	0.13; 16.0		CCL4L1	9560	12	
NFS1	9054	0.18; 4.4		CCL3	6348	11	
PMP22	5376	0.19; 2.1		LAX1	54900	5.8	
PTGER3	5733	0.19; 4.8		AXUD1	64651	3.8	
SFXN1	94081	0.31; 3.6		OSM	5008	3.6	
SOCS1	8651	0.19; 5.1		TMEM88	92162	3.6	
UPP1	7378	0.13; 3.7		CD69	969	3.5	
ECHDC3	79746	3.4; 0.21		CCNA1	8900	3	
GNLY	10578	22; 0.36		MGC4677	112597	2.9	
LGALS12	85329	4.9; 0.21		C5AR1	728	2.8	
RGS1	5996	3.1; 0.13		MCL1	4170	2.8	
TFDP1	7027	2.9; 0.16		NFKBID	84807	2.8	
TMEM71	137835	4.2; 0.31		SGK	6446	2.8	
VNN2	8875	6.2; 0.20		NR4A3	8013	2.7	
				PLCXD1	55344	2.6	
GZMB	3002	136	**>high IL3**	HMGA1	3159	2.5	
IL2RB	3560	48		NR4A2	4929	2.4	
IL1B	3553	31		SOCS2	8835	2.3	
HAL	3034	0.09		BIRC3	330	2.2	
DPEP2	64174	0.05		METRNL	284207	2.2	
RARRES3	5920	0.04		ARHGEF3	50650	0.48	
CXCR4	7852	0.02		HMGN4	10473	0.48	
				HIST1H2AC	8334	0.47	
IL1RL1	9173	6.9	**>moderate IL3**	CP110	9738	0.45	
DPYD	1806	4.5		LMBRD1	55788	0.45	
OBFC2A	64859	3.5		TPRG1L	127262	0.45	
FHL1	2273	0.37		FAM96A	84191	0.44	
				HAGH	3029	0.44	
PRG2	5553	26.0; 3.6	**20 short vs. long**	VNN3	55350	0.44	
FCGR2A	2212	7.5; 1.4	IL-3	ZNF608	57507	0.39	
DUOXA2	405753	7.3; 0.4		MCM7	4176	0.38	
MMP1	4312	7.1; 1.4					
FCGR2B	2213	5.3; 1.6		PRPSAP1	5635	6.8	**16 aIgE(15hr) set**
EMP	2012	5.3; 28.0		ATXN2	6311	5.5	
MRPS6	64968	4.3; 11.0		ABCC5	10057	4.3	
IL1RL1	9173	4.1; 0.38		CA8	767	4.1	
FLJ14123	79899	3.8; 14.0		ZNF439	90594	4.1	
LGALS1	3956	3.0; 15.0		XPO1	7514	3.8	
NGK7	4818	2.1; 8.2		MNDA	4332	3.7	
NEK6	10783	1.1; 5.4		ACVR1	90	3.6	
NCOR2	9612	1.1; 6.5		FAM111A	63901	3.6	
DPYSL2	1808	0.94; 7.7		RASA1	5921	3.6	
LAGE3	8270	0.88; 6.5		TTC15	51112	3.6	
LRP5L	91355	0.35; 0.09		ARHGAP19	84986	3.3	
FFAR2	2867	0.31; 3.5		CDC16	8881	3.2	
S100P	6286	0.2; 0.04		KIAA0240	23506	3.2	
LGALS12	85239	0.18; 0.05		NFKB2	4791	0.28	
HIST1H1C	3006	0.14; 1.0		TNFAIP3	7128	0.22	

Table A3
